# Inhibition of the assembly of *Plasmodium* Hsp70-1 and Hsp40 complex blocks DNA replication by destabilizing ribonucleotide reductase subunit-2

**DOI:** 10.1128/mbio.02129-25

**Published:** 2025-09-12

**Authors:** Iman Alkhatib, Deepanshu Garg, Wahida Tabassum, Km Tanishka, Mrinal Kanti Bhattacharyya, Sunanda Bhattacharyya

**Affiliations:** 1 Department of Biotechnology and Bioinformatics, School of Life Sciences, University of Hyderabad 28614, Hyderabad, Telangana, India; 2 Department of Biochemistry, School of Life Sciences, University of Hyderabad 28614, Hyderabad, Telangana, India; The George Washington University Milken Institute of Public Health, Washington, DC, USA

**Keywords:** *Plasmodium *ribonucleotide reductase, replication arrest, *Plasmodium *Hsp70-Hsp40, benzo-hydroxamate, 116-9e

## Abstract

*Plasmodium falciparum* undergoes endoreduplication, in which its nuclear, mitochondrial, and apicoplast genomes are replicated multiple times without cytokinesis,; enabling replication as a promising target for arresting parasite growth and pathogenicity. Previously, it was reported that the inhibition of expression of small subunit of ribonucleotide reductase (PfR2) leads to the inhibition of DNA synthesis and growth of the parasites. Here, we report the molecular determinant that is necessary for aiding early folding intermediates of PfR2. We find that PfR2 interacts with the cytosolic Type-I Hsp40-cochaperone PfYdj1 (Pf3D7_1437900), which subsequently interacts with PfHsp70-1 through its Histidine-Proline-Aspartate (HPD) motif. Such association of PfR2 is specific toward PfYdj1 and not with other cytosolic Type II Hsp40-cochaperone PfSis1 (PF3D7_0213100). We show that perturbation of association between PfYdj1 and PfHsp70-1 by small molecule 116-9e results in the disruption of PfR2 homeostasis within the parasite. As a result, there is a significant reduction in dNTP production leading to a replication arrest. We demonstrate that the combination of 116-9e and benzo-hydroxamate, a catalytic inhibitor of PfR2, works in a synergistic fashion. Together, our work suggests that targeting PfYdj1-PfHsp70-1 complex assembly in the context of ribonucleotide reductase function can be used as an attractive strategy to curb malaria.

**IMPORTANCE:**

Ribonucleotide reductase is an important enzyme which catalyzes the reduction of ribonucleotides to deoxyribonucleotides, and inhibition of the synthesis of its catalytic subunit (PfR2) leads to significant growth inhibition in malaria parasites. Our study deciphers the molecular determinants that are essential for the maturation of PfR2. We show that *Plasmodium* Hsp40 cochaperone, PfYdj1 (Pf3D7_1437900), is the molecular cochaperone that facilitates the binding of PfR2 with PfHsp70-1, which is crucial for PfR2 folding and stability. We show that perturbation of the assembly between PfYdj1-PfHsp70-1 by a small molecule 116-9e destabilizes PfR2 and subsequently inhibits dNTP formation, resulting in replication arrest in the parasite. We demonstrate that 116-9e and the catalytic inhibitor of PfR2, benzo hydroxamate, potentiate each other’s action. Also, the combination of both the inhibitors displays profound synergism in 3D7 parasites. We propose that the combination of 116-9e and benzo hydroxamate can be employed as an attractive anti-malaria strategy.

## INTRODUCTION

Malaria is a vector-borne disease that causes high mortality rate and economic loss. According to the latest report of WHO (1), about 2.2 billion cases of malaria have been reported in 2024, which poses a serious concern. The main challenge in the treatment of malaria is the development of drug-resistant parasites. Presently, combination therapy approaches are being utilized as a promising strategy to overcome drug resistance. Here, we show that inhibition of cellular homeostasis of PfR2 in combination with its catalytic inhibition can be employed as an effective strategy to reduce parasite burden. Ribonucleotide reductase is an important enzyme that catalyzes the reduction of ribonucleotides to form deoxy-ribonucleotides, which are the building blocks of DNA. The holoenzyme is composed of two large R1 subunits and each of the two small subunits R2 and R4 (2). The catalytic pocket of R2 subunit carries a highly oxidized redox center, formed by two ferric ions and a stable tyrosyl radical which is crucial for nucleotide reduction (3). It was demonstrated earlier that antisense oligonucleotide targeting against the translation start site of PfR2 (PF3D7_1405600) leads to significant inhibition of DNA synthesis and growth inhibition in the parasite (4). PfR2 expression is highest in the mature trophozoites, and this subunit is localized in the cytoplasm of the parasite (2). It was reported that benzo-hydroxamate, the catalytic inhibitor of *Plasmodium* ribonucleotide reductase, can inhibit the growth of parasite (IC_50_ = 17 µM) at 20-fold lower doses than that required to inhibit purified human ribonucleotide reductase (IC_50_ = 400 µM) (5). This result suggests that there is a difference between the structures of *Plasmodium* and human ribonucleotide reductases, hence, *Plasmodium* ribonucleotide reductase qualifies as an important anti-malaria target. The molecular determinants that are involved in the stabilization of PfR2 have not been determined before.

Hydroxyurea acts as an inhibitor of ribonucleotide reductase, which prevents the synthesis of daughter strand DNA and thereby causes cell cycle arrest (6). The -NH_2_-OH moiety of hydroxyurea serves as a potent metal chelator and is responsible for scavenging the free radical/metal ions. It inactivates ribonucleotide reductase by reducing the diferric tyrosyl radical present in small subunit-2 *via* one-electron transfer (7, 8) and subsequently inhibits the production of dNTPs. Hydroxyurea displays weak inhibitory effect on *Plasmodium falciparum* Dd2-infected erythrocyte culture with an IC_50_ of about 792 µM, whereas one of its derivative benzo-hydroxamate shows 50-fold reduced IC_50_ value (5).

Hsp70 works synergistically with Hsp40 cochaperones (J domain proteins) and nucleotide exchange factors (NEFs) to aid in native protein folding. Hsp40 cochaperones bind to the hydrophobic patches of newly synthesized polypeptides through their carboxy terminal domain (CTD). It was observed that the members of Hsp40 cochaperones display distinct client sequence selectivity and, thus, regulate client maturation (9). Upon client binding, Hsp40 binds to the ATP-bound form of Hsp70 through their J-domain and activates its ATP hydrolysis and subsequently loads the client to the substrate binding domain of Hsp70. Subsequently, NEFs bind to the ADP-bound Hsp70 and exchange ADP for ATP, which, in turn, causes the release of the folded substrate from Hsp70.

*P. falciparum* harbors four isoforms of Hsp70 and two isoforms of NEFs (Nucleotide exchange factors), of which PfHsp70-1 (PF3D7_0818900) is constitutively expressed in all the erythrocytic stages and is present in the cytoplasm along with PfHsp70-Z, which is the NEF of PfHsp70-1 (10, 11). *P. falciparum* harbors 44 members of Hsp40, of which only 2 cytosolic cochaperones have been demonstrated to activate the ATP hydrolysis of PfHsp70-1. One of them is Type I Hsp40, Pf3D7_1437900 (PfYdj1) (12), which is the yeast ortholog of Ydj1. The other one is a Type-II Hsp40, PF3D7_0213100 (PfSis1) (13), which is the yeast ortholog of Sis1. Previously, both of them were described as Hsp40. In order to distinguish between them, and other members of Hsp40, we have designated them as PfYdj1 and PfSis1 in this paper. The J domain of both the cochaperones consists of four helices, in which the helix II and helix III are joined by a small loop with highly conserved (histidine-proline-aspartate) HPD motif; however, it has not been established whether they interact with PfHsp70-1 through that motif. It has been reported that PfYdj1 is farnesylated during intra-erythrocytic stages (14), and the farnesylation is essential for the survivability of malaria parasite under temperature stress (15).

It has been demonstrated that a small molecule 116-9e, due to the presence of a diphenyl moiety, sterically interferes in the binding of J-domain with prokaryotic Hsp70 (DnaK) (16). As a result, the J-domain protein fails to stimulate the intrinsic ATPase activity of DnaK, which is essential for the interaction of DnaK with unfolded substrates. However, the inhibitory effect of 116-9e is very specific to the DnaJ-DnaK complex and has no such inhibitory effect on DnaK-GrpE (NEF of DnaK) complex association. The effect of 116-9e in *Plasmodium* culture has not been tested so far.

It was reported earlier that Hsp70 cochaperone Ydj1 or its human ortholog physically interacts with the ribonucleotide reductase small subunit 2, and this interaction is important for the stability of ribonucleotide reductase in yeast as well as in human cells (17). In that study, it was observed that the substrate-binding domain (carboxy-terminal domain I and carboxy-terminal domain II) of Ydj1 plays an important role in hydroxyurea-mediated sensitivity in yeast. We wanted to determine whether PfYdj1 is involved in the regulation of PfR2 homeostasis. We also asked whether the other cytoplasmic cochaperone PfSis1 can substitute PfYdj1 during the formation of early folding intermediates of PfR2, as these two cochaperones are cytoplasmic and both have been shown to activate ATPase activity of PfHsp70-1.

In the present study, we have established that PfR2 is a client of PfYdj1. We show that disruption of the PfYdj1-PfHsp70-1 complex assembly by 116-9e destabilizes parasite PfR2 in a dose-dependant manner, which is correlated with the reduction of intracellular dNTP level and S-phase arrest of the parasite. Additionally, we demonstrate that 116-9e sensitizes the parasite toward benzo-hydroxamate inhibition. We propose that the combination of 116-9e and benzo-hydroxamate can be employed as a novel approach to reduce parasite burden.

## RESULTS

### *Plasmodium* Ydj1 genetically complements yeast Ydj1 and bypasses HU-mediated toxicity in yeast

**Fig 1:**
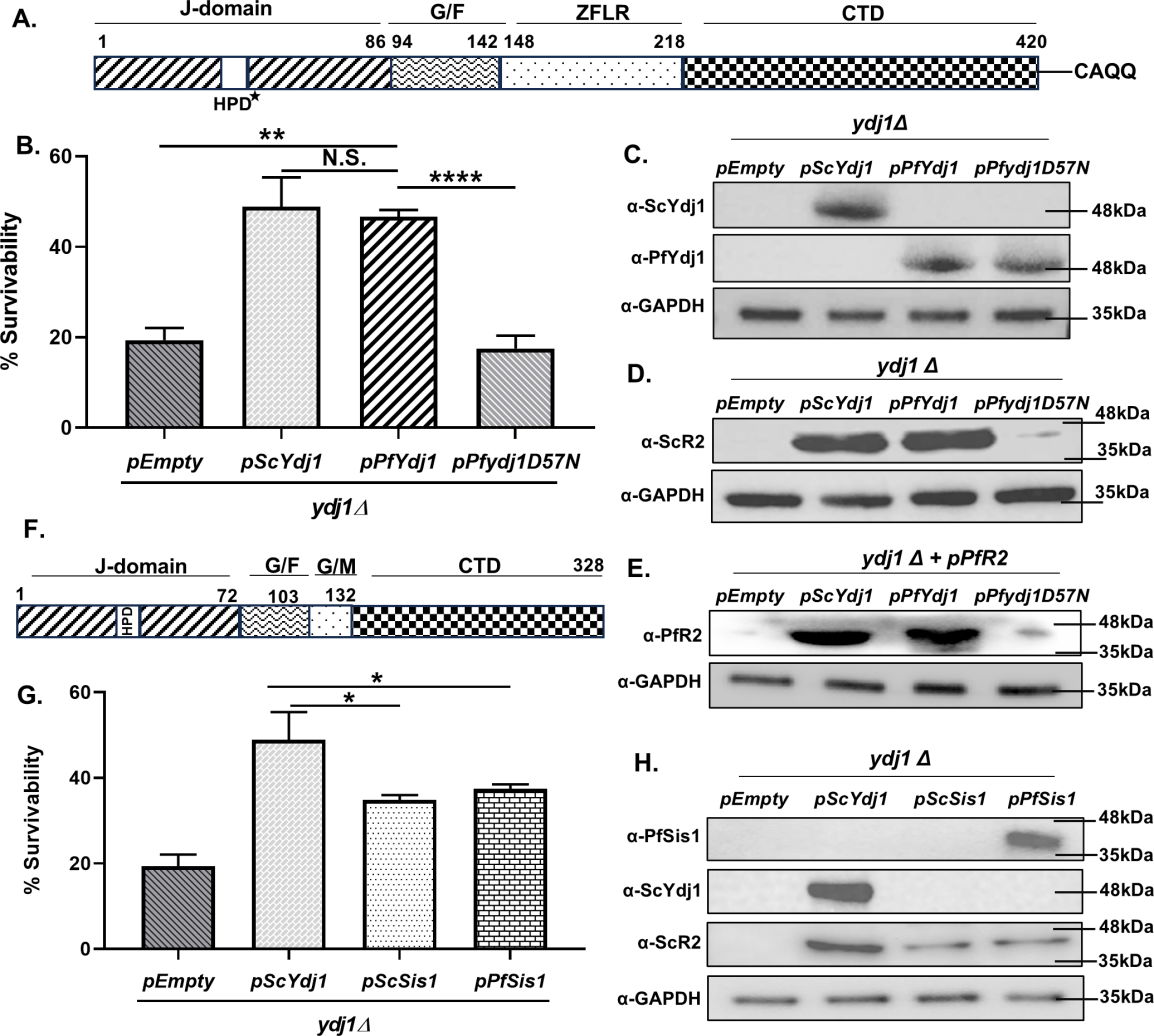
*Plasmodium* Ydj1 genetically complements yeast Ydj1 and bypasses HU-mediated toxicity in yeast: (**A**) Schematic presentation of the key conserved domains of PfYdj1. The D57^*^ mapped within the HPD motif was mutated in our subsequent assays. (**B**) *ydj1Δ* yeast strains harboring empty vector, *pScYdj1*, *pPfYdj1*, or *pPfydj1D57N* were generated and each was treated with 200 mM HU for 16 h. Experiments were repeated for three times with each strain and cell survivability for each strain was plotted on the *Y*-axis. The expression of *PfYdj1* as well as *ScYdj1* rescued the growth of *ydj1Δ*, whereas the *Pfydj1D57N* mutant failed to complement the defect. The mean values ± SD were plotted using GraphPad PRISM 8, *P-*values were calculated according to the two tailed Student’s *t* test (**P < 0.01, NS; not significant, ****P < 0.0001). (**C**) Western blot showing the expression of ScYdj1 as well as PfYdj1 and its mutant in the respective strains. GAPDH served as a loading control. (**D**) Western blot analysis showing comparable expression of ScR2 in strains expressing yeast or *Plasmodium* Ydj1; however, complete destabilization of ScR2 occurred in the *Pfydj1D57N* mutant-expressing strain. (**E**) PfR2 was ectopically expressed in the above-mentioned strains, and its expression was determined by western blot analysis. We observed that PfR2 protein is stable in *ScYdj1*/*PfYdj1-*expressing strains; however, the protein is destabilized in *Pfydj1D57N* mutant-expressing strain. (**F**) Schematic representation of PfSis1 domain organization, which does not possess Zn finger-like region as that in Ydj1, rather it has an additional region, rich in glycine/methionine residues. (**G**) *ydj1Δ* yeast strains harboring; empty vector, *pScYdj1*, *pScSis1*, or *pPfSis1* were generated and individually treated with 200 mM HU for 16 h. The experiments were repeated three times with each strain, and we observed that the expression of neither *ScSis1* nor *PfSis1* could rescue the growth of *ydj1Δ* strain, as that of *ScYdj1*. The mean values ± SD were plotted, *P*-values were calculated using the two-tailed Student’s *t* test (*P < 0.05). (**H**) Western blot analysis showing the expression of ScYdj1 and PfSis1 in the respective *ydj1Δ* strains. We observe that ScYdj1-mediated endogenous stabilization of ScR2 can’t be compensated by ScSis1 or PfSis1.

To decipher whether PfYdj1 regulates the proteostasis of PfR2 subunit and thereby aids in the function of ribonucleotide reductase, we employed yeast as a surrogate model system. Previously, it was reported that ScYdj1 (*Saccharomyces cerevisiae*), an ortholog of PfYdj1, regulates the function of yeast ribonucleotide reductase, and mutation in the substrate-binding domain of ScYdj1 makes the cells sensitive to hydroxyurea (HU) (17). The pairwise alignment of the CTD of ScYdj1 (17) and that of PfYdj1 (18) showed sequence conservation (43.5% sequence similarities) between the substrate-binding domains of the two orthologs (Fig. S1A). The schematic representation (Fig. 1A) shows the domain organization of PfYdj1, which harbors a J-domain at the amino-terminal end, followed by a region rich in glycine and phenylalanine residues. The middle domain has a Zinc finger-like region (ZFLR), and at the end of the carboxy-terminal domain (CTD), there is a CAQQ motif. To establish whether PfYdj1 serves as a potential regulator of ribonucleotide reductase, we performed hydroxyurea sensitivity assay. We used *ydj1Δ* strain and transformed a centromeric expression vector harboring *PfYdj1*. As a positive control, we transformed centromeric vector-expressing *ScYdj1,* and as a negative control, we transformed empty vector in *ydj1Δ* strain, respectively. We have generated a point mutant of *Pfydj1D57N* in the J-domain as shown in the schematic presentation, in which the aspartic acid is mutated to asparagine within the HPD motif (Fig. 1A). We generated an isogenic strain which expresses a single copy of *Pfydj1D57N* mutant in *ydj1Δ* strain. We subjected each of the strains to 200 mM HU for 16 h and observed that *PfYdj1* can rescue the HU-mediated toxicity in *ydj1Δ* strain to the same level as that of *ScYdj1-*expressing strain (Fig. 1B). However, the *Pfydj1D57N* mutant harboring strain failed to do so and scored similar survivability as that of the negative control. To rule out the possibility that loss of reversal of HU-mediated toxicity in *Pfydj1D57N* is due to the lack of the expression of the mutant protein, we monitored the mutant protein expression. We observed comparable expression of the wild-type and mutant protein (Fig. 1C). To that end, we generated antibody against PfYdj1 by purifying the parasite protein within bacterial system; as the protein was copurified with a bacterial protein of 35 kDa, we extracted the recombinant PfYdj1 from the polyacrylamide gel and generated the antibody against the protein (Fig. S2A). We find that the polyclonal antibodies for yeast and *Plasmodium* orthologs have no cross reactivity, as seen in the western blot that anti-ScYdj1 does not recognize PfYdj1 and vice versa (Fig. 1C; Fig. S2B). Next, we wanted to understand whether the phenotype observed in the mutant strain is linked with the loss of endogenous stability of ScR2. We probed the endogenous level of ScR2 using antibodies against the human R2 which cross-reacted with ScR2 (Fig. 1D). We observed that *PfYdj1-*expressing strain stabilizes ScR2 same as that expressing ScYdj1; however, there is a complete absence of ScR2 in *Pfydj1D57N* harboring strain, same as that harboring the empty plasmid. To investigate whether the similar effect happens on PfR2 stability, we ectopically expressed PfR2 in above-mentioned strains and probed for PfR2 level using antibody against PfR2 that was generated in the lab (Fig. S2C). Our study shows that while PfR2 is stabilized in *PfYdj1*-expressing strain, it could not be stabilized within the *PfD57Nydj1* mutant strain (Fig. 1E). Thus, our study concludes that PfYdj1 is involved in PfR2 homeostasis in an Hsp70-dependent manner.

It was earlier reported that PfR2 shows distinct cytoplasmic foci in all the intra-erythrocytic stages of the parasite (2). *Plasmodium* genome harbors 44 members of Hsp40 out of which 2 of them, PfYdj1 and a PfSis1, have been shown to be present in parasite cytosol (12, 13). Schematic representation of PfSis1 shows that it possesses the J-domain, followed by G/F and G/M region and lacks the ZFLR in its carboxy-terminal domain (Fig. 1F). We tested whether PfSis1 can substitute the function of PfYdj1 toward the stabilization of PfR2. To that end, we generated two new *ydj1Δ* strains, one harboring a centromeric expression vector with *PfSis1* and the other harboring its yeast ortholog *ScSis1* and subjected them to HU as before. The survivability data show that both *ScSis1-* and *PfSis1-*expressing strains cannot rescue HU-mediated toxicity similar to that of *ScYdj1* (Fig. 1G); however, there is a partial complementation as their survivability score is little better than that of *ydj1Δ* strain. When we probed for the endogenous level of ScR2 in the above-mentioned strain, it was evident that *PfSis1-* or *ScSis1-*expressing strains could not provide the stability of ScR2 as that of *ScYdj1-*expressing strain (Fig. 1H). To show the expression of PfSis1 in the yeast system, we purified PfSis1 from the bacterial system and generated antibody against the recombinant protein (Fig. S2D). Together, our study indicates that PfYdj1 has a distinct function in homeostasis of ribonucleotide reductase subunit 2 and its activity, which is not compensated by PfSis1. Although PfYdj1 and PfSis1 cochaperones share 51.6% sequence similarities in their J-domain (Fig. S1B), PfSis1 cannot functionally substitute PfYdj1.

### *Plasmodium* Pfydj1^D57N^ mutant fails to interact and activate the ATPase activity of PfHsp70-1

**Fig 2:**
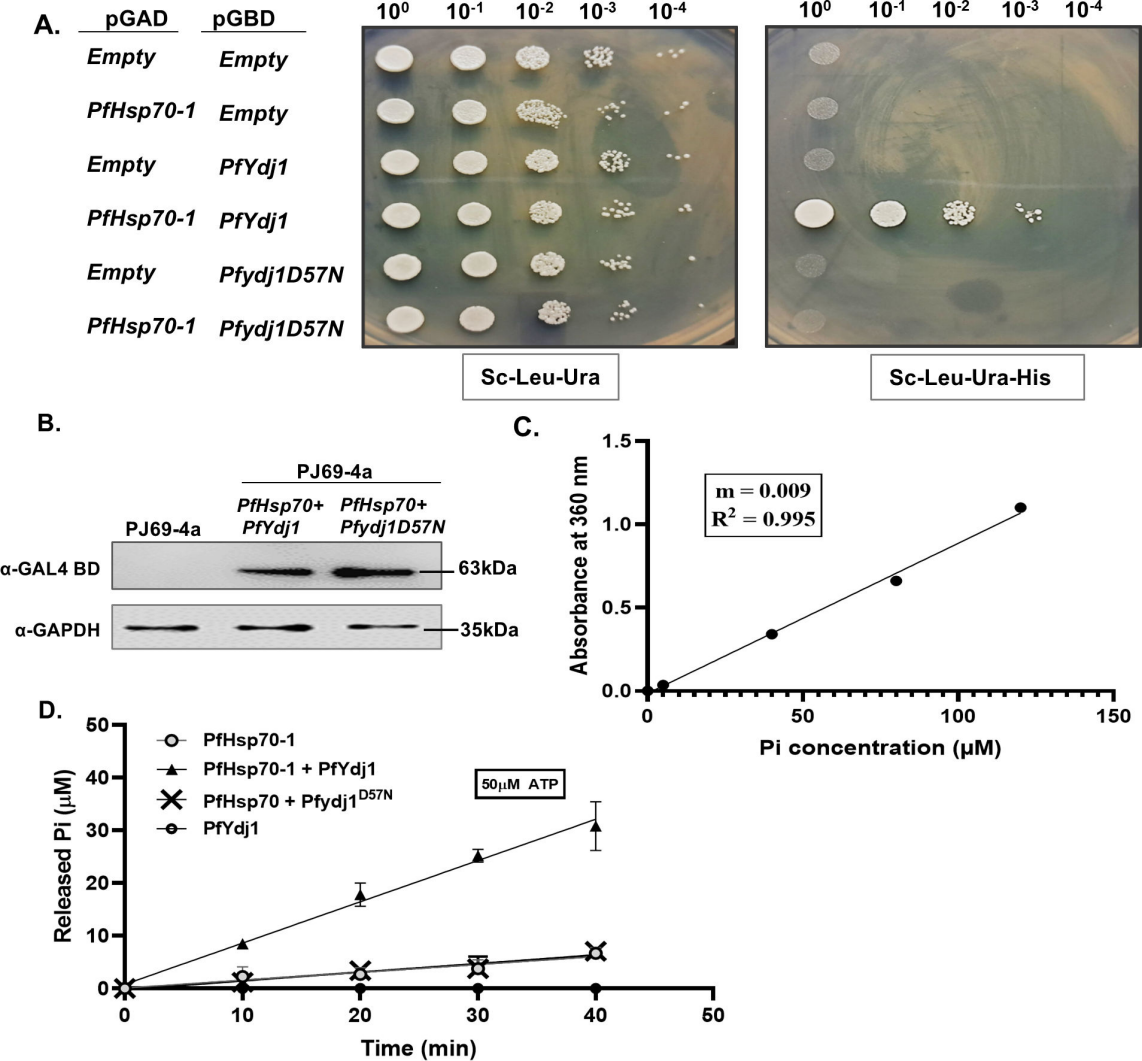
*Plasmodium* hsp40^D57N^ mutant fails to interact and activate the ATPase activity of PfHsp70-1: (**A**) Left-panel shows the schematic presentation of the bait and prey constructs that were transformed to PJ694A strain to score for the protein-protein interaction utilizing the reporter gene *HIS3*. Yeast two-hybrid assay shows that PfHsp70-1 interacts with PfYdj1, but not with its mutant Pfydj1^D57N^. (**B**) Immunoblot analysis showing the expression of PfYdj1 and *PfYdj1^D57N^* fused to the GAL4 DNA-binding domain (GBD) in respective yeast strains. The wild-type PJ69-4A strain served as a negative control. GAPDH was used as a loading control. (**C**) Standard curve for Pi quantification in the ATPase assay. A linear relationship was observed between Pi concentration and absorbance at 360 nm, with a slope of 0.009 and *R*² = 0.995. This curve was used to calculate Pi released in ATPase reactions. (**D**) ATP hydrolysis stimulation of PfHsp70-1 by PfYdj1 measured using the EnzChek phosphate assay kit. Reactions were performed with 50 µM ATP and 2 µM of each protein. Conditions tested included PfHsp70-1 alone (filled gray circle), PfHsp70−1 + PfYdj1 (▲), PfHsp70−1 + *PfYdj1^D57N^* (cross, ×), and PfYdj1 alone (●). Inorganic phosphate (Pi) release was measured over time, using a slope factor of 0.009. Data represent mean ± SD of three independent replicates.

To understand the mechanism behind the loss of function phenotype of *Pfydj1D57N* mutant, we wanted to probe the interaction between mutant Pfydj1^D57N^ and PfHsp70-1 using yeast two-hybrid analysis. To that end, we cloned the wild-type *PfYdj1* and its mutant individually in the bait vector, as a fusion of GAL4 DNA-binding domain and cloned *PfHsp70-1* in the prey vector, as a fusion of *GAL4* activation domain and studied their interaction in PJ69-4A strain (Fig. 2A). Earlier, we have shown that PfYdj1 displays a physical association with PfHsp70-1 in a yeast two-hybrid assay (19). We found that PfYdj1 and PfHsp70-1 together can activate the *HIS3* reporter gene expression; as a result, the strain harboring both the plasmids can grow in triple dropout media (Fig. 2A, fourth row). However, there is no growth for the mutant Pfydj1^D57N^ and Hsp70-1 combination, indicating that aspartate at the 57th position is essential to mediate the interaction with Hsp70-1 (sixth row). To rule out the possibility that the loss of interaction of the mutant is due to its loss of expression in heterologous system, we isolated the total protein from the strains and probed for the expression of Pfydj1^D57N^ using the antibody against GAL4 DNA-binding domain (Fig. 2B). We found comparable expression of the wild-type and mutant proteins in both strains. Next, to establish whether the loss of interaction between Pfydj1^D57N^ with PfHsp70-1, indeed, results in the loss of stimulation of the ATPase activity of PfHsp70-1, we purified mutant protein Pfydj1^D57N^ (Fig. S2E) and performed the ATP hydrolysis assay. To rule out the possibility of any DnaK contamination from bacteria, we performed western blot analysis using anti-DnaK antibody. We found that PfHsp70-1, PfYdj1, and Pfydj1^D57N^ are devoid of DnaK contamination (Fig. S3A). To measure ATPase activity, we used EnzChek Phosphate assay kit (Molecular Probes). In this assay, the enzyme PNP (purine nucleoside phosphorylase) can convert MESG (2-amino-6-mercapto-7-methyl-purine riboside), to ribose 1-phosphate and 2-amino-6-mercapto-7-methylpurine in the presence of inorganic phosphate, and this conversion leads to the shift in absorbance from 330 nm to 360 nm. The increase in the absorbance is directly linked with the increase in phosphate concentration as seen in the standard curve (Fig. 2C). We incubated purified PfHsp70-1 with 50 µM ATP at 37°C for 40 min to allow ATP hydrolysis. At different time intervals, a small aliquot was transferred to another reaction mixture carrying MESG and PNP, and the absorbance was measured at 360 nm. The amount of phosphate generated at different time interval was calculated by dividing the absorbance with the slope of the standard curve 0.009 (Fig. 2C). We find that PfHsp70-1 showed a basal level of ATPase activity (Fig. 2D). Purified PfYdj1 itself does not possess any ATPase activity on its own but can stimulate the ATPase activity of PfHsp70-1 (Fig. 2D). We measured the rate of ATP hydrolysis of PfHsp70-1 in the presence of PfYdj1 as 0.391 µM min^−1^ per µM concentration of the enzyme. However, the mutant Pfydj1^D57N^ fails to activate the ATPase activity of PfHsp70-1, and in the presence of the mutant protein, the rate of ATP hydrolysis of PfHsp70-1 has dropped down to 0.083 µM min^−1^ per µM concentration of the enzyme (Fig. 2D). To rule out the possibility that the purified proteins are having bound ATP as contaminants, we measured the ATPase activity of each protein in the absence of ATP and did not find any phosphate generated as shown in Fig. S3B. Together, our study establishes that to manifest the cochaperone activity of PfYdj1, its association with Hsp70-1 is essential and a single mutation in the HPD motif can abrogate its interaction completely; as a result, the mutant fails to stimulate ATPase activity of PfHsp70-1.

### Association between PfYdj1 and PfHsp70-1 is abrogated by 116-9e

**Fig 3:**
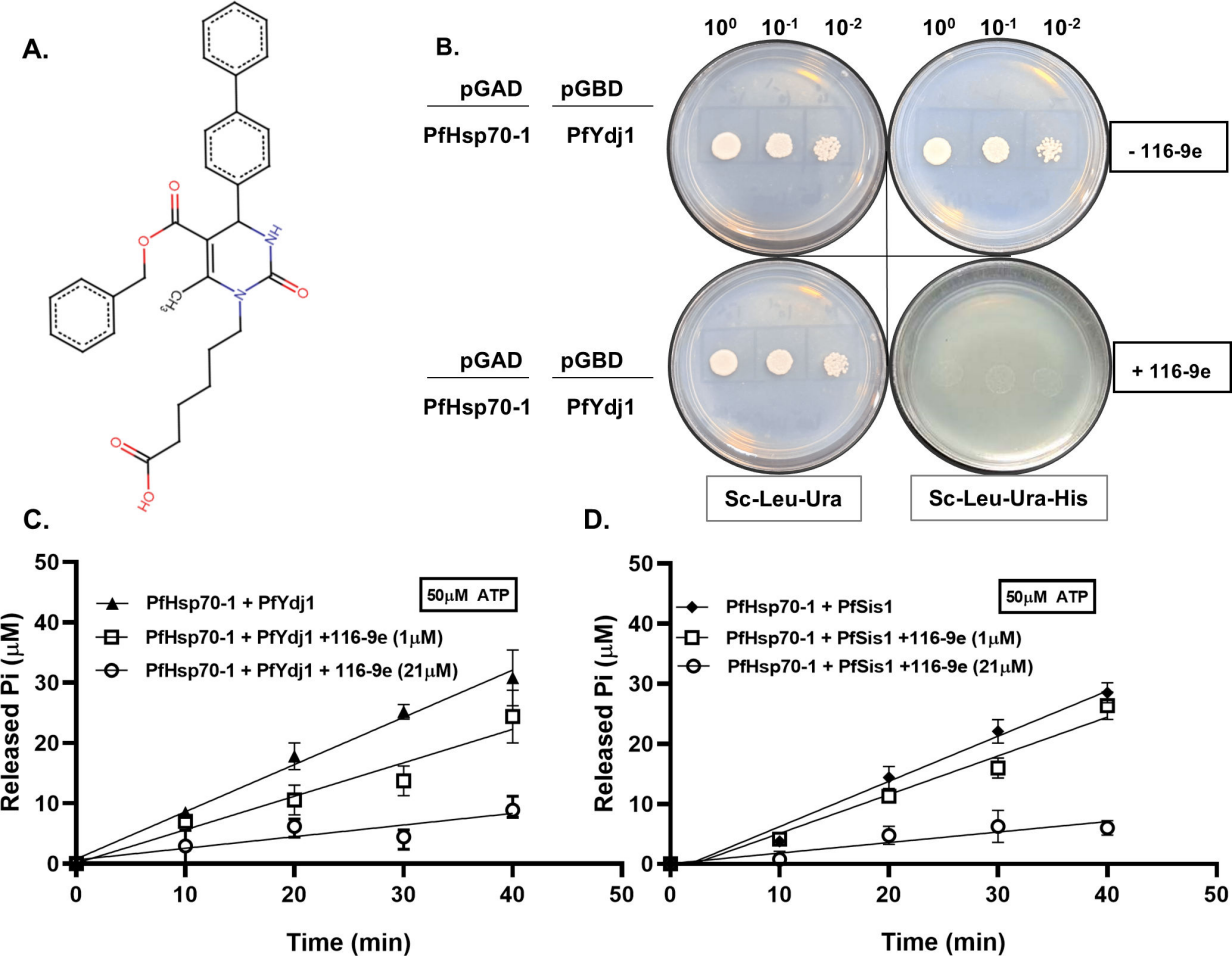
Association between PfYdj1 and PfHsp70-1 is abrogated by 116-9e: (**A**) Chemical structure of 116-9e, highlighting its diphenyl groups. (**B**) Left panel shows the schematic presentation of the bait and prey constructs that were transformed to PJ694A strain to score for the protein-protein interaction in the absence and presence of 116-9e. There was no growth on 116-9e added triple-drop out plates, indicating that 116-9e inhibits the association between PfHsp70-1 and PfYdj1. (**C**) ATP hydrolysis stimulation of PfHsp70-1 by PfYdj1 in the presence and absence of 116-9e. The assay was performed using the EnzChek phosphate assay kit with 50 µM ATP and 2 µM of each protein. The tested conditions included PfHsp70−1 + PfYdj1 without 116-9e (▲), in the presence of 1 µM 116-9e (□), and in the presence of 21 µM 116-9e (○). Inorganic phosphate (Pi) release was measured over time, using a slope factor of 0.009. Bars represent the mean of three independent experiments. (**D**) ATP hydrolysis stimulation of PfHsp70-1 by PfSis1 in the presence and absence of 116-9e. The assay was performed using the EnzChek phosphate assay kit with 50 µM ATP and 2 µM of each protein. The tested conditions included PfHsp70−1 + PfSis1 without 116-9e (♦), in the presence of 1 µM 116-9e (□) and in the presence of 21 µM 116-9e (○). Inorganic phosphate (Pi) release was measured over time, using a slope factor of 0.009. Bars represent the mean of three independent experiments.

Earlier, it was shown that the addition of a small-molecule 116-9e (Fig. 3A) causes suppression of the J-domain-mediated ATP hydrolysis of bacterial DnaJ-DnaK complex by 80% (16). We wanted to study the effect of that molecule on the interaction of *Plasmodium* Hsp70-1 and PfYdj1. We performed the yeast two hybrid analysis to score their interaction in the presence of 116-9e. We used 116-9e containing triple drop out plates and observed that the interaction between PfYdj1 and PfHsp70-1 was completely abrogated in such condition (Fig. 3B). Furthermore, we measured the ATP hydrolysis of the above-mentioned pair in the presence of 116-9e. We added two different concentrations (1 µM and 20 µM) of 116-9e in the reaction buffer and observed a reduced stimulation of PfYdj1-mediated ATP hydrolysis of PfHsp70-1 in a dose-dependent manner (Fig. 3C). In the presence of 1 µM 116-9e, PfYdj1-induced rate of ATP hydrolysis of PfHsp70-1 was determined as 0.277 µM min^−1^ per µM concentration of the enzyme, whereas in the presence of 20 µM 116-9e, the same was reduced to 0.096 µM min^−1^ per µM concentration of the enzyme. Earlier, it was shown that PfSis1 can stimulate the ATPase activity of PfHsp70-1 (13). In our assay condition, we find that at 50 µM ATP concentration, ATP hydrolysis of PfHsp70-1 in the presence of PfSis1 as 0.37 µM min^−1^ per µM concentration of the enzyme. We have tested that PfSis1 is devoid of bacterial DnaK or any ATP contaminants (Fig. S3C and D, respectively). We wanted to determine whether 116-9e can inhibit the ATPase activity of the PfSis1-PfHsp70-1 pair. We found a similar kind of inhibition in the PfSis1-mediated stimulation of PfHsp70-1 ATPase activity (Fig. 3D). In the presence of 1 µM 116-9e, PfSis1-induced rate of ATP hydrolysis of PfHsp70-1 was reduced to 0.322 µM min^−1^ per µM concentration of the enzyme, whereas in the presence of 20 µM 116-9e, the same was further reduced to 0.087 µM min^−1^ per µM concentration of the enzyme. Thus, our study confirms that 116-9e can disrupt the J-domain interaction of PfYdj1/PfSis1 with PfHsp70-1 and subsequently inhibits the ATPase activity of PfYdj1/PfSis1-PfHsp70-1 complex.

### PfR2 physically associates with PfYdj1 but not with PfSis1

**Fig 4:**
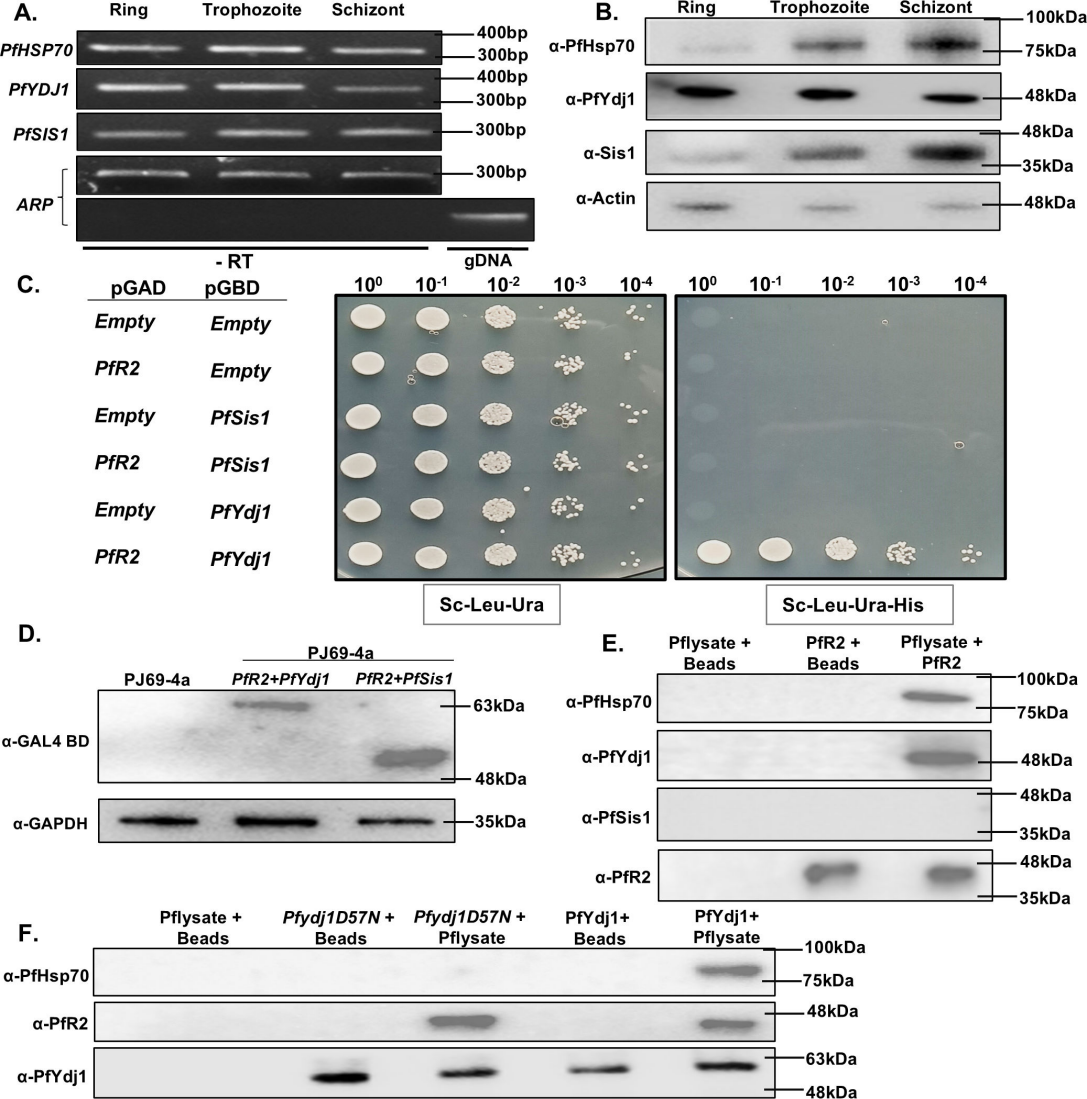
PfR2 physically associates with PfYdj1 but not with PfSis1: (**A**) Reverse transcription-polymerase chain reaction (RT-PCR) analysis shows the stage-specific expression of PfHsp70-1, PfYdj1, and PfSis1 in various asexual developmental stages of *P. falciparum*. Gene-specific primers amplified 305 bp, 350 bp, and 299 bp fragments from cDNA. Aspartate-rich protein (ARP) (300 bp) served as a loading control. -RT control confirmed no genomic DNA contamination, and gDNA control validated primer specificity. (**B**) Western blot analysis confirms the abundant expression of PfHsp70-1, PfYdj1, and PfSis1 in trophozoite stage of the parasites, actin served as a loading control. (**C**) Left-panel shows the schematic presentation of the bait and prey constructs that were transformed to PJ694A strain to score for the protein-protein interaction utilizing the reporter gene *HIS3*. Yeast two-hybrid assay showing the interaction of PfR2 with PfYdj1, but not with PfSis1. (**D**) Western blot showing PfYdj1 and PfSis1 expression as GAL4-GBD fusions in yeast. GAPDH served as a loading control. (**E**) His-tagged PfR2 was immobilized on Ni-NTA beads and incubated with *P. falciparum* trophozoite stage-specific lysate. Co-purification assay identifies the stable PfR2-PfHsp70-PfYdj1 complex formation. (**F**) Reverse-co-purification assay was performed in which purified PfYdj1 and purified Pfydj1^D57N^ were bound with Ni-NTA beads and trophozoite stage-specific parasite lysates were allowed to bind with the above-mentioned protein bound-beads. PfR2 of parasite was detected in both cases, but PfHsp70 was observed to be co-purified only with wild-type PfYdj1 but not with the mutant. The negative controls were used as parasite lysate bound with beads and purified PfYdj1/Pfydj1^D57N^ bound with beads.

To decipher the molecular determinants that are involved in early folding intermediates of PfR2, we investigated whether PfYdj1 or PfSis1 can interact with PfR2. As it was reported that both PfYdj1 and PfSis1 are cytoplasmic, we first tested whether both are expressed in the trophozoite stage, where the expression of PfR2 was reported to be maximum (2). The stage-specific expression data confirm that PfHsp70-1 along with PfYdj1 and PfSis1 are abundantly present at both the RNA and protein level during the replicative stage of the parasite (trophozoite and schizont) (Fig. 4A and B, respectively). We used yeast two-hybrid analysis to check the physical association between PfR2 with each of the two above-mentioned cochaperones. To that end, *PfR2* was cloned in the prey vector, whereas *PfYdj1* and *PfSis1* were cloned individually in the bait vector. We tested the *HIS3* reporter gene activation as a readout of the interaction between PfR2 and cochaperones and observed that PfR2 interacts with PfYdj1 (Fig. 4C, sixth row) but fails to interact with PfSis1 (Fig. 4C, fourth row). The loss of interaction was not due to less expression of PfSis1, as western blot shows similar levels of expression of both the cochaperones, when probed with antibody against GAL4 DNA binding domain (Fig. 4D). We employed an independent approach to determine their interaction. We purified full length PfR2 (Fig. S2F) as a histidine-tagged protein and allowed it to bind to the Ni-NTA chromatographic column. We allowed the trophozoite stage-specific parasite lysate to pass through the column to examine whether PfYdj1 or PfSis1 was copurified with PfR2. The experiment was repeated with three independent batches of synchronized trophozoite-stage-specific parasites, and we observed that PfYdj1 and PfHsp70-1 were copurified with PfR2 (Fig. 4E). The presence of PfSis1 was not detected in the complex although PfSis1 was expressed in the trophozoite stage (Fig. 4B). Further to validate the physical association between PfR2 and PfYdj1, we did reverse co-purification assay. Typically, we allowed the binding of purified PfYdj1 or Pfydj1^D57N^ mutants with Ni-NTA chromatographic columns and trophozoite-specific parasite lysate were passed through the column. We observed that PfR2 was copurified with both PfYdj1 and Pfydj1^D57N^ (Fig. 4F). However, as expected, PfHsp70-1 was not detected in Pfydj1^D57N^ cochaperone-PfR2 complex, which might be due to disruption in the association between PfHsp70-1 and Pfydj1^D57N^. We have found that none of the antibodies used in the copurification assay are cross reacting with the host proteins (Fig. S3E). Together, we conclude that PfR2 specifically interacts with PfYdj1 but not with PfSis1. We reason it might be due to a large variation in the substrate-binding domains of the two cochaperones (25.9% sequence similarities) (Fig. S4A). Furthermore, mutation in single amino-acid within the HPD motif of PfYdj1 disrupts the association of PfR2-PfYdj1 complex with PfHsp70-1.

### Treatment with 116-9e leads to PfR2 destabilization and concomitant reduction in dNTP formation within the parasite

**Fig 5:**
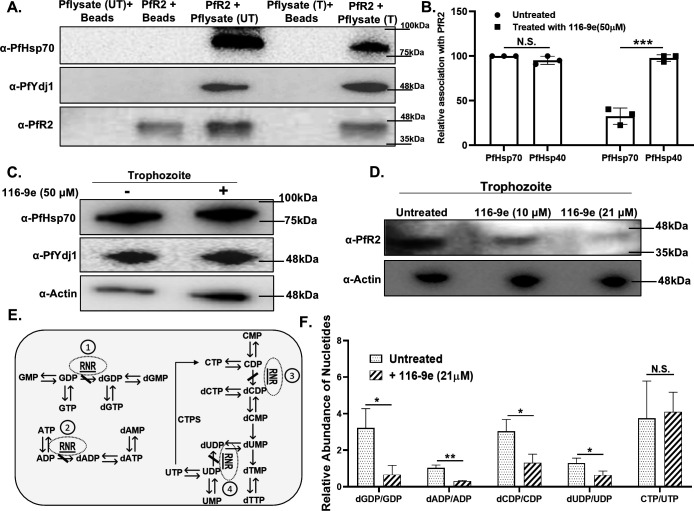
Treatment with 116-9e leads to PfR2 destabilization and concomitant reduction in dNTP formation within the parasite: (**A**) Co-purification assay was performed in which His-tagged PfR2 was immobilized on Ni-NTA beads and incubated with *P. falciparum* trophozoite lysate; one batch was grown with 50 µM 116-9e for 24 h and other batch was untreated parasite lysate. Western blot analysis indicates that PfR2 interaction with PfYdj1 remains unaffected under both conditions, whereas a significant reduction in PfHsp70-1 binding with PfR2-PfYdj1 complex is observed in 116-9e treated parasite. The experiment was repeated with three independent batches of parasites. (**B**) The relative association of PfHsp70 and PfYdj1 with PfR2 was calculated by normalizing signal intensities to the untreated condition (set as 100%). Data represent mean ± SEM from three independent experiments. While PfYdj1 binding to PfR2 was not affected by 116-9e treatment, a significant reduction in PfHsp70 association was observed. Statistical analysis was performed using two-tailed Student’s *t* test (***P < 0.001 , N.S., not significant). (**C**) Western blot analysis showing the level of PfHsp70-1 and PfYdj1 in treated and untreated parasite samples. Actin served as loading control. (**D**) Parasite proteins were extracted from trophozoite stage-specific untreated culture and also from two sets of parasite cultures that were preincubated with either 10 µM or 25 µM of 116-9e for 24 h. Western blot analysis showing a dose-dependent reduction in the endogenous level of PfR2 in 116-9e-treated samples compared to untreated. Actin served as loading control. (**E**) Schematic representation of PfR2-mediated dNTP synthesis. The diagram illustrates the metabolic pathways through which PfR2 catalyzes the production of dNTP. Inhibition of RNR leads to the accumulation of ribonucleotide substrates (GDP, ADP, CDP, UDP) and a decrease in the production of respective deoxyribonucleotides (dGDP, dADP, dCDP, dUDP). (**F**) LC-MS-based metabolic analysis was done, and levels of 10 nucleotides dUDP, UDP, dADP, ADP, dCDP, CDP, dGDP, GDP, CTP, and UTP were quantified in two independent batches of 21 µM 116-9e treated and that of untreated *P. falciparum* parasites. The ratios of dGDP/GDP, dADP/ADP, dCDP/CDP, dUDP/UDP, and CTP/UTP were analyzed to assess the metabolic impact of 116-9e treatment. The CTP/UTP ratio was used as a negative control, as its interconversion is independent of PfR2 activity. The mean values ± SD were plotted; *P*-values were calculated using the two-tailed Student’s *t* test (*P < 0.05, **P < 0.01, NS; not significant).

As our biochemical assay shows that 116-9e inhibits the stimulation of the ATPase activity of PfHsp70-1 by PfYdj1, we postulate that the effect of 116-9e might be detrimental for PfR2 homeostasis due to an inhibition of association between PfR2-PfYdj1 and PfHsp70-1. We wanted to determine whether similar inhibition is seen in the assembly of cochaperone-chaperone complex (PfHsp70-1-PfYdj1) in 116-9e treated 3D7 parasites. For that, we took synchronized late-ring stage-specific parasites and divided them into two parts, while one part was treated with 50 µM 116-9e for 24 h, other part was grown without the chemical and used as an untreated control. Subsequently, we harvested both sets of parasites and prepared parasite lysate once they reached the late-trophozoite stage. We used nickel NTA beads that is prebound with purified PfR2 protein and allowed 116-9e treated or untreated parasite lysate to bind PfR2. We observed that PfR2 association with PfYdj1 remained unperturbed in both the conditions; however, there is a significant reduction in binding of PfHsp70-1 in the treated sample (Fig. 5A). The experiment was repeated three times with new batches of treated and untreated parasites. We estimated the band intensities of PfHsp70-1, PfYdj1, and PfR2 by image J software and found that although the relative association of PfYdj1 with PfR2 remained unaltered, however, the relative association of PfHsp70-1 with PfR2 is reduced by threefold under 116-9e treated condition (Fig. 5B). To rule out the possibility that 116-9e dose used in our assay does not reduce the expression of PfHsp70-1, we compared the levels of PfHsp70-1 and PfYdj1 in treated and untreated parasite samples and found them to be comparable (Fig. 5C). Thus, our study concludes that 116-9e perturbs the *in vivo* complex formation of PfHsp70-1-PfYdj1 as well. We wanted to decipher whether the inhibition of PfHsp70-1-PfYdj1 complex assembly affects PfR2 homeostasis within the parasite. We measured the endogenous level of PfR2 in 116-9e treated parasite samples. We treated synchronized late-ring stage parasites with two increasing doses of 116-9e, namely 10 µM and 21 µM, harvested parasites at the late trophozoite stage and extracted total protein. We observed there is a reduction in the stability of PfR2 in the 116-9e treated sample in a dose dependent manner (Fig. 5D). We measured the activity of PfR2 in 21 µM 116-9e treated parasites. The schematic diagram (Fig. 5E) represents the metabolic pathways through which PfR2 generates the dNTPs. We prepared parasite lysates grown under untreated and 21 µM 116-9e treated condition and isolated the total metabolites from both of them. Using LC/MS analysis, we estimated the levels of 10 metabolites dGDP, GDP, dADP, ADP, dCDP, CDP, dUDP, UDP, CTP, and UTP (20) from the treated and untreated parasites and measured the ratio of dGDP/GDP, dADP/ADP, dCDP/CDP, dUDP/UDP, and CTP/UTP. We anticipate that loss of activity of PfR2 will eventually cause an increase in its substrate concentration, i.e., GDP/ADP/CDP/UDP, along with a subsequent decrease in product concentration. Hence, the ratio of product/substrate will reduce in PfR2 inhibitory condition. We repeated the experiments with two more biological replicates and observed a significant reduction in dGDP/GDP, dADP/ADP, dCDP/CDP, and dUDP/UDP ratio in 21 µM 116-9e treated parasites (Fig. 5F). We measured the ratio of two metabolites CTP/UTP in which UTP is converted to CTP in PfR2 independent pathway. We do not find any significant difference in the ratio of CTP/UTP in 116-9e treated condition, which acts as a negative control of our assay. Together, our study confirms that 116-9e treatment, by virtue of inhibiting PfYdj1-PfR2-PfHsp70-1 complex assembly, destabilizes PfR2 homeostasis and causes a significant reduction in the dNTP pool of the parasites.

### Treatment of 116-9e or benzo-hydroxamate cause replication arrest in the asexual stage of *P. falciparum*

**Fig 6:**
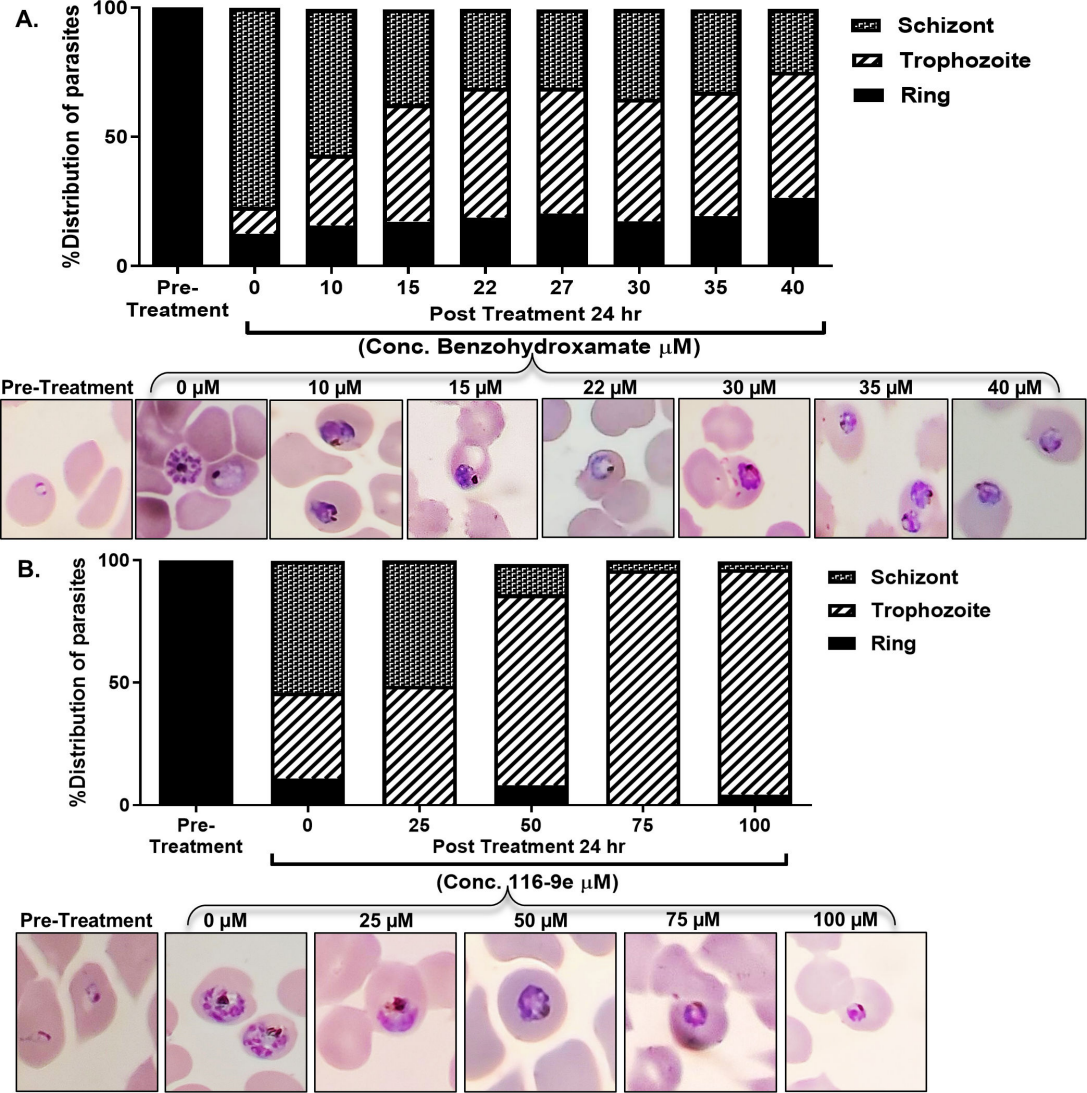
Treatment of 116-9e or benzo-hydroxamate causes replication arrest in the asexual stage of *P. falciparum*: (**A**) The stacked bar graph showing the percentage distribution of *P. falciparum* parasites at ring, trophozoite, and schizont stages following 24 h treatment with increasing concentrations of benzo-hydroxamate (0–40 µM). A clear developmental arrest was observed at the trophozoite stage in a dose-dependent manner. The experiment was repeated independently five times under identical conditions, and the mean values from all replicates are plotted. Giemsa-stained blood smears were prepared at 24 h post-treatment, and representative images are shown below the graph. Representative Giemsa-stained fields were selected from more than 100 imaged fields per condition to illustrate the typical morphology observed at each concentration. (**B**) The stacked bar graph showing the effect of 116-9e treatment (0–100 µM) on parasite stage distribution after 24 h. Like benzo-hydroxamate, 116-9e caused arrest at the trophozoite stage in a concentration-dependent manner. The experiment was repeated three times independently, and plotted values represent the mean. Giemsa-stained images from 24 h time points are shown below, selected from >100 observed fields per condition to reflect the most representative parasite morphology.

Earlier, it was shown that benzo hydroxamate (a derivative of hydroxy urea) displayed antimalarial activity in *P. falciparum* Dd2 strain with an IC_50_ of 17 µM (5). We investigated the effect of benzo hydroxamate on *Plasmodium* replication. We used late-ring stage-specific parasite culture and divided it into eight parts, while one part was used as untreated control, other seven parts were exposed to increasing doses of benzo hydroxamate for 24 h as shown in the figure (Fig. 6A). At the end of the incubation period, we counted the distribution of rings, trophozoites, and schizonts in each case. We plotted the percent distribution of parasites on the *Y*-axis for each dose of benzo hydroxamate as shown in the *X*-axis. We observed that while the untreated parasites were majorly converted to schizonts at the end of 24 h, there was a sharp decrease in the percentage of schizont stage of the parasites with increasing dose of benzo hydroxamate. We found that parasites were majorly arrested at the trophozoite stage, i.e., replicative stage of their development. This is expected due to the inhibition of PfR2 enzymatic activity by benzo hydroxamate. We found that the treated parasite-trophozoites show enlarged morphology as shown in the bottom panel. We wanted to investigate whether a similar kind of replication arrest happens in 116-9e treated 3D7 parasites. To that end, we used late-ring stage-specific parasite culture and treated them with increasing doses of 116-9e for 24 h. We plotted the percent distribution of parasites in the *Y*-axis for each dose of 116-9e as shown in *X*-axis. We found that 116-9e treatment also causes replication arrest in the parasites, same as that of benzo hydroxamate. We observed that with exposure to 50 µM 116-9e or above, there was a significant decrease in the schizont stage of the parasites, and parasites were mostly arrested at the trophozoite stages (Fig. 6B). However, when we investigate the morphology of the treated parasites, amoeboid-shaped trophozoites were observed even at 25 µM 116-9e, which corroborates with the reduction of PfR2 abundance and activity at this concentration (Fig. 5D and F). As these were counted as trophozoites, the ratio of trophozoite to schizont remains unchanged at 25 µM 116-9e doses. Together, we conclude that either the inhibition of PfR2 homeostasis or the catalytic inhibition of PfR2 results in similar kind of developmental defect in the parasite.

### Inhibition of Hsp40-Hsp70-1 complex assembly sensitizes the parasite toward ribonucleotide reductase inhibitors

**Fig 7:**
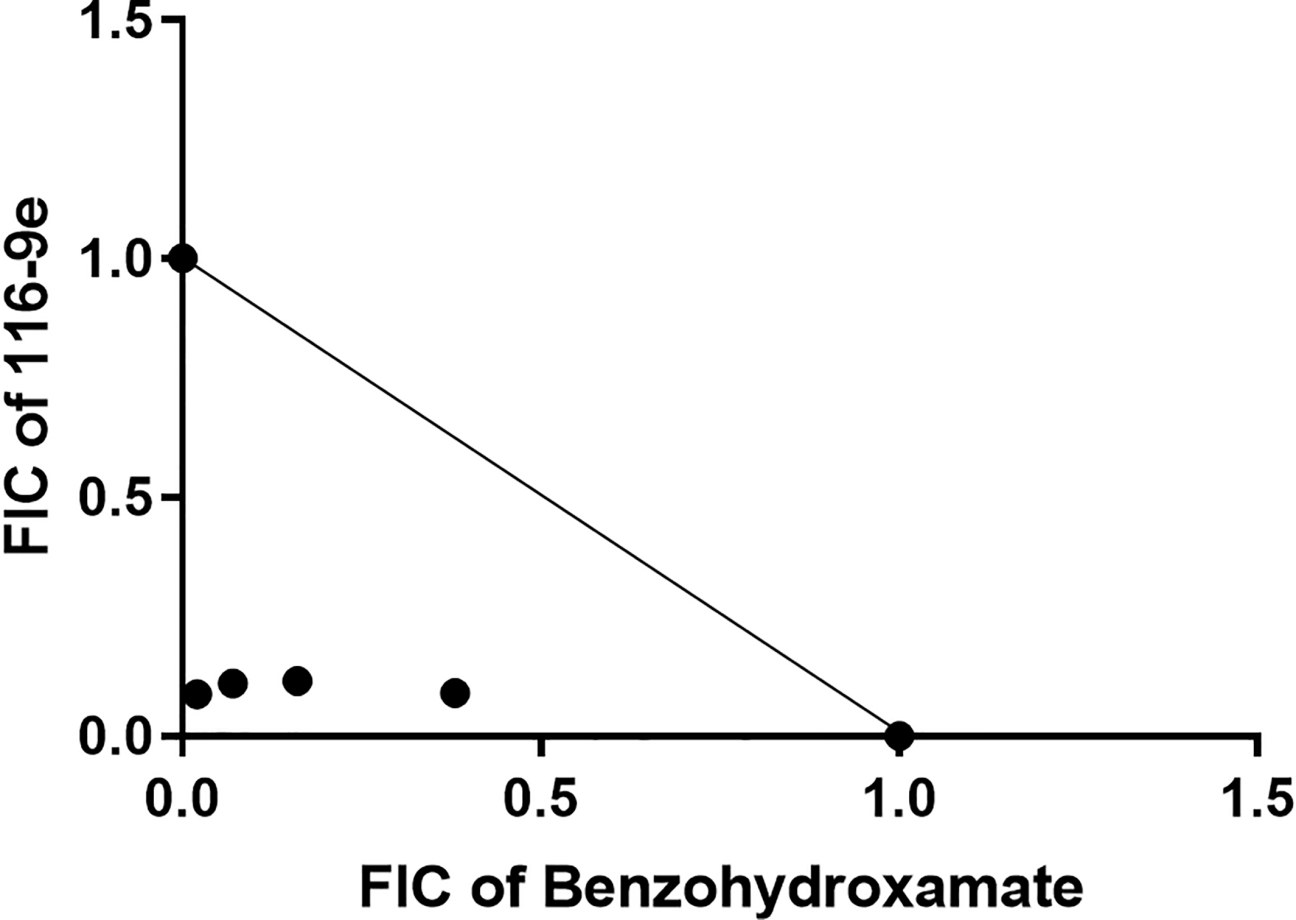
Inhibition of Hsp40-Hsp70-1 complex assembly sensitizes the parasite towards ribonucleotide reductase inhibitors: Synergistic interaction between the PfR2 inhibitor benzo hydroxamate and 116-9e was observed by plotting an isobologram of benzo hydroxamate and 116-9e in 3D7 parasite. Each point represents the mean half-maximal inhibitory concentration (IC_50_) of the drug combinations. A solid line was drawn between the IC_50_ values of each drug when used alone. FIC, fractional inhibitory concentration.

Since benzo hydroxamate inhibits the catalytic activity of PfR2 and 116-9e inhibits the proteostasis of PfR2, we predicted that destabilization of PfR2 due to the action of 116-9e would render the parasites more sensitive toward benzo hydroxamate and *vice-versa*. We determined IC_50_ of 116-9e as well as benzo hydroxamate in 3D7 parasites. Typically, synchronous ring-stage-specific parasites were treated with different concentrations of the respective drugs for 48 h, percent inhibition was plotted against different concentrations, and IC_50_ values were determined. The IC_50_ values for 116-9e and benzo hydroxamate were found as 21.723 µM and 23.85 µM, respectively, in our assay condition (Table 1). Next, we incubated the 3D7 parasites with different concentrations of 116-9e in the presence of IC_50_ concentration (23.85 µM) of benzo hydroxamate. It was observed that the presence of benzo hydroxamate could lower the IC_50_ of 116-9e to 0.39 µM. Hence, benzo hydroxamate potentiates the inhibitory effect of 116-9e by a factor of 55.7 (Table 1). We next investigated whether 116-9e could also potentiate the action of benzo hydroxamate. To that end, we treated the 3D7 parasites with various doses of benzo hydroxamate in the presence of IC_50_ doses of 116-9e (21.723 µM). We observed that the IC_50_ value of benzo hydroxamate was dropped to 0.34 µM, resulting in 70.15-fold potentiation of benzo hydroxamate by 116-9e (Table 1). In order to understand whether this kind of interaction is specific between these two drugs, we checked the effect of well-established antimalarial drug Chloroquine on the IC_50_ of benzo hydroxamate/116-9e. We incubated 3D7 parasites with increasing doses of 116-9e, along with IC_50_ concentration of chloroquine (26.4 nM), which was earlier determined in our laboratory condition (21). IC_50_ values of 116-9e in such combination were observed to be not altered much and were 20.57 µM. Similar kind of assays was done by treating 3D7 parasites with increasing doses of benzo hydroxamate in combination of IC_50_ doses of chloroquine, and we found the IC_50_ value of benzo hydroxamate not altered much and was 19.8 µM. Thus, we found that chloroquine can’t potentiate the inhibitory action of neither 116-9e nor benzo hydroxamate (Table 1). We further wanted to investigate whether the interaction between 116-9e and benzo hydroxamate is additive or synergistic. To that end, we performed a fixed-ratio drug combination assay. We determined the mean fractional inhibitory concentration (FIC) values for each combination assay from their respective dose-responsive curve as shown in Table 2. The FICs were plotted in isobologram (Fig. 7). We found that the combination of 116-9e and benzo hydroxamate displays a synergistic interaction within each other. Thus, our study indicates that the combination of inhibition of PfR2 homeostasis and inhibition of PfR2 enzymatic activity can reduce the parasite burden in an effective manner.

**TABLE 1:** Potentiation of benzo hydroxamate action by 116-9e and *vice versa*

Strain	Combination of drugs	IC_50_ (µM)	Potentiation factor
3D7	116-9e (alone)	21.723	1
	116-9e (benzo hydroxamate)	0.39	55.7
	Benzo hydroxamate (alone)	23.85	1
	Benzo hydroxamate (116-9e)	0.34	70.15
	116-9e (CQ)	20.57	1.02
	Benzo hydroxamate (CQ)	19.8	1.2

**TABLE 2:** FIC values drug combinations^*a*^

Strain	Benzo hydroxamate: 116-9e	FIC of Benzo hydroxamate	FIC of 116-9e	∑FIC
	5:0	1	0	1
	4:1	0.38	0.09	0.47
3D7	3:2	0.16	0.115	0.27
	2:3	0.07	0.110	0.18
	1:4	0.02	0.088	0.1
	0:5	0	1	1

^*a*^ FIC, fixed inhibitory concentration.

## DISCUSSION

**Fig 8:**
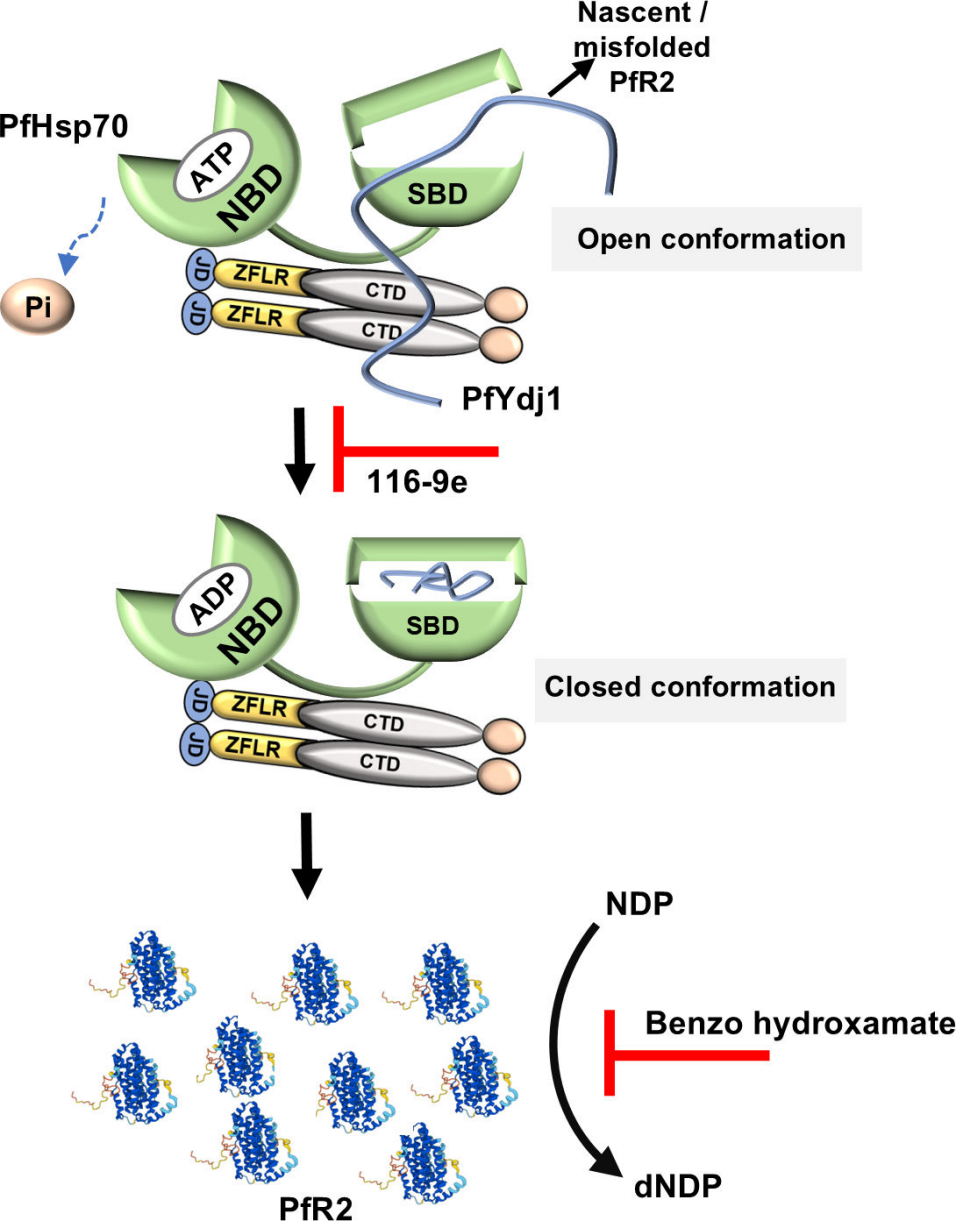
Dual targeting of PfR2 disrupts stability and dNDP production in malaria parasites: Disrupting PfR2 through a dual-targeting strategy interferes with its stability and function, leading to an insufficient supply of dNDPs. The small molecule 116-9e blocks the interaction between PfHsp70-1 and PfYdj1, preventing the proper folding and stabilization of PfR2 and benzo hydroxamate inhibits the catalytic activity of PfR2.

Our present study provides promising evidence that targeting the PfHsp70-1-PfYdj1 assembly can be utilized as a novel approach to destabilize ribonucleotide reductase subunit-2 of malaria parasite. An earlier study indicated the essential function of PfYdj1 in the asexual stage of the parasite (15), the mutagenesis fitness score (MFS) was reported as −2.43 for PfYdj1 (22). In order to establish the specificity of PfYdj1 in maintenance of PfR2 homeostasis, we incorporated another Type II Hsp40 cochaperone PfSis1 in our analysis. First, we have demonstrated that PfR2 is specifically associated with PfYdj1 but not with PfSis1. Second, in yeast surrogate system, we have established that PfYdj1 but not PfSis1 is required to stabilize ScR2, thereby essential to rescue HU-mediated toxicity in yeast. Third, utilizing both *in vitro* and *in vivo* analysis, we have shown that 116-9e-mediated inhibition of PfHsp70-1-PfYdj1 complex assembly destabilizes PfR2. We reason that PfYdj1 binds to the unfolded PfR2 through its substrate-binding domain and, subsequently, transfers the client to PfHsp70-1 for further folding. The binding of J domain of PfYdj1 with PfHsp70-1 is critical in stimulating the ATP hydrolysis of the chaperone, which is essential for the release of folded client. A point mutation at the HPD motif of PfYdj1 or the addition of 116-9e inhibits the homeostasis of R2 subunit. Fourth, we demonstrate that 116-9e treatment reduces the effective conversion of NTPs to dNTPs as a result causes replication arrest of the parasite. Finally, we demonstrate that the combination of 116-9e and the catalytic inhibitor of PfR2 can reduce parasite burden in an effective manner. Thus, our study proposes a novel anti-malaria strategy, which is well illustrated by the schematic diagram (Fig. 8).

Our study underlines the importance of specificity of the carboxy-terminal domain of PfYdj1 in binding to PfR2, which precedes the binding of PfYdj1 with PfHsp70-1. Carboxy-terminal domain of PfSis1 is considerably different than that of PfYdj1 due to the absence of ZFLR region and the presence of additional glycine/methionine-rich region (18). Our yeast two-hybrid assay and copurification assay fail to detect association between PfSis1 with PfR2. Our study demonstrates that although the J-domains of PfSis1 share considerable sequence similarities with PfYdj1 and 116-9e can inhibit the ATPase stimulation of PfHsp70-1 driven by both PfSis1 and PfYdj1, binding of PfR2 nascent polypeptide to PfYdj1 is the deciding factor for its folding. Thus, our study opens up new research avenues to identify the interaction sites between PfYdj1-PfR2. Understanding the molecular parameters involved in such interactions will help to design better inhibitors for PfR2 folding.

The differential inhibition of *Plasmodium* and human ribonucleotide reductase by benzo hydroxamate indicates that PfR2 catalytic pocket might be structurally different than its human ortholog, and hence, it can be a potential anti-malaria target. However, benzo hydroxamate does not qualify as a potent inhibitor of PfR2 due to its moderately high IC_50_ value (23.85 µM). Our study shows that the addition of IC_50_ dose of 116-9e increases the effectiveness of benzo-hydroxamate by several folds and drastically reduces its IC_50_. We show that this kind of potentiation is specific for 116-9e and could not be observed for other anti-malaria drug, such as Chloroquine. Similarly, our study shows benzo hydroxamate also can potentiate the action of 116-9e by several folds in 3D7 parasites. The profound synergism between 116-9e and benzo hydroxamate indicates that the inhibition of the assembly of PfHsp70-1-PfYdj1 may destabilize other replication proteins in the parasites, and it will be interesting to evaluate further the entire clientele of PfYdj1.

Thus, our research has opened new avenues in malaria research which emphasizes that targeting chaperone-cochaperone assembly along with the functional inhibition of crucial replication protein of the parasite can be employed as an efficient strategy to combat malaria. It will be interesting to decipher whether PfR2 requires additional folding by the Hsp90-chaperone system, and in such case, unraveling the Hsp90-cochaperone plasticity in maintaining PfR2 homeostasis can result in the identification of novel anti-malaria target.

## MATERIALS AND METHODS

### Plasmids

Full-length *PfYdj1*, *PfSis1*, *PfR2*, *PfHsp70-1,* and *ScSis1* were individually amplified from 3D7 Trophozoite stage-specific c-DNA using the primer pairs OSB 605 (5′ GACGGATCCATGTTTTTCTCATCAGGTTTTCC 3′)-OSB 286 (5′ GAAGTCGACTTATTGTTG AGCACAAGCTACTC 3′), OSB 746 (5′ GCCGGATCCATGGGGAAGGATTATTATTC 3′)-OSB 747 (5′ GCCGTCGACTTAGAATGTATTTGCCAATGTTTC 3′), OSB 587 (5′ GATGGATCCATGGCTGATGTTATAAACATTTCTAG 3′)-OSB 588 (5′ GATGTCGACTTAAAATTCCGTATTCAGACAAAAG 3′), OSB 219 (5′ GCAGGATCCATGGCT AGTGCAAAAGGTTC 3′)-OSB 220 (5′ GCAGTCGACTTAATCAACTTCTTCAACTGTTG 3′) and OSB 705 (5′ GACGGATCCATGGTCAAGGAGACAAAAC 3′)-OSB 749 (5′ GCCGTCGACTTAAAAATTTTCATCTATAGCAG 3′), respectively. Each primer pairs are having a BamHI site incorporated in the forward primer and SalI restriction endonuclease site incorporated in the reverse primer. *PfYdj1* and *PfSis1* were cloned individually in *pGBDUC1* vector (23) and confirmed by sequencing. Both were further subcloned to yeast expression vector *pRS313* (24) and the bacterial expression vector *pET28a* (25). Full-length *PfR2* as well as *PfHsp70-1* were cloned to *pGADC1* (23) and confirmed by sequencing. Both the ORFs were separately subcloned to *pET28a* vector as well. Further *PfR2* and *ScSis1* were subcloned within yeast expression vectors *pLA* (26) and *pRS313,* respectively. *ScYdj1* cloned in *pRS313* was already available in the laboratory. The sequence encoding truncated PfR2^210-340^ was amplified from 3D7 c-DNA using the forward primer OSB 682 (5′ CCAGGATCCATGAATAAATTACACGGTTTGACATTTAG 3′) having BamHI site and the reverse primer OSB 588 and cloned into *pET28a* and further confirmed by sequencing.

### Site-directed mutagenesis

Point mutation (D to N) was generated at the 57th amino acid of *PfYdj1* by mutating the codon AAA to AGA using the splice-overlap-extension (SOE) PCR technique. To insert the point mutation at *Pfydj1D57N*, the coding sequence was amplified in two segments. The first segment was amplified by using the primer pair OSB 605 and OSB 670 (5′ CTTTTCTGGATCACCTCCTTTATTTGGATGATGAATAAT AG 3′), and the second segment was amplified using the primer pair OSB 671 (5′ AAGCTAGCTATTATTCATCATCCAAATAAAGGAGGTGATCC 3′) and OSB 286. Subsequently, using the two PCR products as a template, full-length *PfYdj1D57N* was amplified using the primer pair OSB 605 and OSB 286 and cloned into the *pRS313* vector. The generation of mutation was confirmed by DNA sequencing. It was further subcloned to *pGBDUC1* and *pET28a* vectors separately.

### Yeast strains

The yeast strains used in this study are listed in Table S1. For the hydroxyurea (HU) sensitivity assay, we transformed empty *pRS313*, *pRS313/ScYdj1, pRS313/PfYdj1, pRS313/Pfydj1D57N, pRS313/ScSis1,* and *pRS313/PfSis1* plasmids individually into the *Δydj1* parental strain to generate *IA1, IA2, IA3, IA4, IA5,* and *IA6,* respectively. Strains *IA7, IA8,* and *IA9* were obtained by sequentially transforming *pLA/PfR2 to IA2, IA3,* and *IA4,* respectively. For yeast two-hybrid (Y2H) analysis, strains *IA10* through *IA19* were generated by introducing the following combinations of bait and prey plasmids; *pGBDUC1-pGADC1*, *pGBDUC1/PfYdj1-pGADC1*, *pGBDUC1-pGADC1/PfHsp70*, *pGBDUC1/PfYdj1-pGADC1/PfHsp70*, *pGBDUC1/Pfydj1D57N-pGADC1*, *pGBDUC1/Pfydj1D57N-pGADC1/PfHsp70*, *pGBDUC1-pGADC1/PfR2, pGBDUC1/PfYdj1-pGADC1/PfR2*, *pGBDUC1/PfSis1-pGADC1*, and *pGBDUC1/PfSis1-pGADC1/PfR2* into the PJ69-4A parental strain.

### *Plasmodium falciparum* culture

The *Plasmodium falciparum* 3D7 strain was cultured at a 5% haematocrit level in RPMI 1640 medium (Himedia) and supplemented with 0.5% (wt/vol) Albumax (Thermo Fisher Scientific) and 0.005% (vol/vol) hypoxanthine (Sigma). The parasites were incubated at 37°C using the candle jar method. Their growth was routinely assessed through microscopic examination of Giemsa-stained smears. To synchronize cultures at the ring stage, a 5% sorbitol (Sigma, St. Louis, MO, USA) treatment was applied, following established protocols (24). Synchronized parasites were collected at different stages: Ring at 18–20 h post-invasion (hpi), Trophozoite at 30–32 hpi, and schizont at 44–45 hpi. The developmental stages of parasites were further confirmed based on their morphology in Giemsa-stained smears examined under a microscope (24).

### Protein purification

Recombinant PfYdj1: The expression vector *pET28a: PfYdj1* having N-terminal His6 tag was transformed into *E. coli* BL21(DE3). The whole transformation mixture was added to 10 mL LB media containing kanamycin and incubated at 37°C overnight. Next morning, 5% of the primary culture was added to 100 mL fresh LB media (containing kanamycin) until the OD_600_ reached 0.6; protein was induced by 1 mM IPTG (Sigma) and grown on Luria broth for 16 h at 16°C. The induced cells were pelleted at 12,000 rpm for 10 min at 4°C. Protein was purified utilizing standard protocol available in the laboratory (25). Briefly, cells were resuspended in a lysis buffer comprising 20 mM phosphate buffer pH 7.6, 300 mM NaCl, 5 mM imidazole, 1 mM PMSF (phenylmethylsulphonyl fluoride), 1% Triton X-100, and 0.25  mg mL^−1^ lysozyme and then subjected to sonication at a 10/20 s ON/OFF cycle with 35% amplitude. Following the pelleting of cell debris at 11,000 rpm for 30 min at 4°C, the supernatant was kept for binding with pre-equilibrated Ni-NTA agarose (Qiagen) beads and then washed with buffers containing 30 mM and 50 mM imidazole. The proteins were eluted at gradient imidazole concentrations (75, 150, and 300  mM). The purest form of PfYdj1 was eluted at 300 mM imidazole concentration. The eluted protein was resolved in 10% SDS-PAGE and visualized by Coomassie brilliant blue R-250 (Bio-Rad). The eluted fraction was pooled and dialyzed against the dialysis buffer containing 20 mM phosphate buffer pH 7.6, 300 mM NaCl, 5% glycerol, and 1 mM DTT. The concentration of the purified protein was determined by UV absorbance at 280 nm by using the extinction coefficient of 25,330 M^−1^ cm^−1^ as calculated from the amino acid sequence by using ExPaSy Protparam tool. The recombinant *Pfydj1D57N* was purified utilizing the same protocol.

The recombinant PfHsp70-1 and PfSis1: The expression vectors harboring pET28a: PfHsp70 and pET28a: PfSis1 having N-terminal His6 tag were transformed into *E. coli* Rosetta (DE3) separately. Induction of both the protein expression was done by adding 1 mM IPTG for 4 h at 37°C. We followed the similar steps for protein purification as mentioned above, and both the proteins were eluted at 300 mM imidazole concentration. The concentration of the purified PfHsp70 and PfSis1 was determined by UV absorbance at 280 nm by using the extinction coefficient of 38,850 M^−1^ cm^−1^ and 34,840 M^−1^ cm^−1^, respectively. The recombinant PfR2 and its truncated version were expressed with 0.5 mM IPTG for 4 h at 37°C, and the protein was eluted at 300 mM imidazole concentration. The concentration of the purified protein was determined by UV absorbance at 280 nm by using the extinction coefficient of 46,410 M^−1^ cm^−1^. The truncated *PfR2*^210–349^ protein was expressed under denaturing conditions using 8 M urea (Qualigens) and induced with 0.5 mM IPTG (Sigma). Typically, IPTG-induced cells were sonicated, and the pellet was resuspended in a solubilization buffer consisting of 25 mM Tris-HCl (pH 8.0), 500 mM NaCl, and 8 M urea, followed by incubation on ice for 30 min. The lysate was then clarified by centrifugation at 12,000 rpm for 45 min at 4°C. The resulting supernatant was incubated with pre-equilibrated Ni-NTA agarose (Qiagen) beads to facilitate protein binding. The resin was subsequently washed with buffers containing 10 mM, 30 mM, and 50 mM imidazole, all supplemented with 8 M urea. The protein was eluted in fractions using 250 mM imidazole and dialyzed against a buffer containing 25 mM Tris-HCl (pH 8.0), 500 mM NaCl, 5% glycerol, 1 mM DTT, and 4 M urea. The concentration of the purified protein was determined by measuring UV absorbance at 280 nm, using an extinction coefficient of 9,970 M⁻¹ cm⁻¹, calculated from the amino acid sequence via the ExPASy ProtParam tool. This truncated purified protein was used for antibody generation.

### Generation of antibodies

Polyclonal antibodies against PfYdj1 and PfSis1 were generated using a standard immunization protocol (27). Briefly, 100 µg of purified protein was diluted in phosphate-buffered saline (PBS) and emulsified in an equal volume of Complete Freund’s Adjuvant (CFA) (Sigma-Aldrich). The antigen-adjuvant emulsion was administered to mice *via* intraperitoneal injection (100 µL), distributed across two sites (50 µL per site). Booster immunizations were administered after 2 weeks using 100 µg of antigen diluted in PBS and emulsified in an equal volume of Incomplete Freund’s Adjuvant (IFA). Two additional booster doses were given at regular intervals. Fourteen days after the final immunization, blood samples (~200 µL) were collected via retro-orbital bleeding. Serum was prepared and analyzed for antigen-specific antibody titer.

Polyclonal antibodies against truncated PfR2 were raised in rabbits following the same immunization protocol as for PfYdj1 and PfSis1. However, for rabbit immunizations, 1 mg of protein was used per immunization boost.

### ATPase assay

The enzymatic activity of PfHsp70-1 was evaluated by measuring its ATP hydrolysis rate using the standard protocol used earlier in our laboratory (25), employing the EnzChek Phosphate Assay Kit (Molecular Probes, E-6646). Briefly, reactions were set up with 2 µM PfHsp70-1 in the presence of 2 µM PfYdj1, PfSis1, or Pfydj1^D57N^ protein in the presence of 50 µM ATP containing reaction buffer (50 mM Tris-HCl, pH 7.5, 2 mM MgCl_2_). The reaction mixtures were incubated at 37°C (±116-9e), and samples were collected at 0, 10, 20, 30, and 40 min. At each time point, aliquots were transferred to a purine nucleoside phosphorylase (PNP) reaction mix at 22°C, as per the manufacturer’s guidelines. During ATP hydrolysis, inorganic phosphate was released and subsequently reacted with MESG (2-amino-6-mercapto-7-methylpurine riboside), leading to the formation of 2-amino-6-mercapto-7-methylpurine, which exhibits absorbance at 360 nm. The rate of ATP hydrolysis was measured from the slope obtained from the regression analysis, which was divided by the total enzyme concentration.

### Yeast two-hybrid analysis

The yeast two-hybrid assay was performed as previously described (26) to assess protein-protein interactions. Briefly, *Saccharomyces cerevisiae* strain PJ69-4A was co-transformed with bait and prey plasmids and selected on synthetic complete (SC) medium lacking leucine and uracil (SC-Leu⁻Ura⁻) to maintain both plasmids. An overnight culture of the transformants was grown at 30°C, followed by dilution into fresh SC-Leu⁻Ura⁻ medium and incubation until mid-log phase. Serial dilutions of the culture were then spotted onto SC-Leu⁻Ura⁻ plates (to verify plasmid maintenance) and SC-Leu⁻Ura⁻His⁻ plates (to assess interaction-dependent *HIS3* reporter activation). The plates were incubated at 30°C for 4 days, after which growth on SC-Leu⁻Ura⁻His⁻ plates was examined as an indicator of protein-protein interaction.

### dNTP isolation and LC-MS

A 20 mL synchronized ring stage-specific *Plasmodium falciparum* 3D7 parasites with a 6% parasitemia were divided into two parts; one part of 10 mL parasites was labeled as untreated control and the other part of 10 mL parasites was treated with 21 µM 116-9e, and both the batches were allowed to grow for 24 h until they reach late trophozoite stage as observed under microscope. Metabolites were extracted from parasite cultures following an adapted protocol (20). In brief, synchronized cultures were lysed using saponin to isolate parasites, after which metabolites were extracted by adding 10 volumes of 0.5 M perchloric acid to parasite pellets. The mixture was vortexed and incubated on ice for 20 min. The extracts were then neutralized with 2.5 M potassium hydroxide and further incubated on ice for 20 min. Following centrifugation at 16,000 × *g* for 15 min at 4°C, the supernatants were filtered using Amicon Ultra (0.5 mL-10 kDa) centrifugal filters at 16,000 × *g* for 15 min at 4°C. The resulting filtrate (~100 µL) was immediately frozen in liquid nitrogen and stored at −80°C for further analysis. Liquid chromatography-mass spectrometry (LC-MS) analysis was performed using a Shimadzu LCMS-8045 triple quadrupole mass spectrometer coupled with a Shimadzu Prominence-I HPLC system. A Shim-pack GIST C18 column (75 mm × 4.0 mm, 3 µm) was used for chromatographic separation at a flow rate of 0.8 mL/min. The mobile phase consisted of Solvent A (0.04% acetic acid in water) and Solvent B (100% acetonitrile). The column oven was maintained at 30°C, and the sample cooler was set at 15°C. The gradient elution was as follows: 100%–80% A from 0–8 min, 80%–20% A from 8–15 min, 20% A from 15–18 min, 20%–80% A from 18–22 min, 80%–100% A from 22–24 min, 100% A from 24–26 min. A sample volume of 10 µL was injected for each run. Electrospray ionization (ESI) was conducted in both positive and negative ionization modes under nebulizing gas flow (3 L/min), heating gas flow (10 L/min), interface temperature of 300°C, drying gas flow was 10 L/min, and desolvation line (DL) temperature of 250°C. Full-scan mass spectra were acquired over an *m*/*z* range of 50–2,000. Raw LC-MS data were processed using MZmine. Peaks were detected, deconvoluted, and aligned using automated algorithms, followed by gap filling. Metabolites were annotated using the KEGG compound database (https://www.genome.jp/kegg/compound/) with a mass tolerance of 0.001–0.05 Da, and the final data were exported in CSV format for analysis.

### RNA isolation and reverse transcription-polymerase chain reaction analysis

Total RNA was extracted from synchronized *P. falciparum* 3D7 parasites at the ring, trophozoite, and schizont stages using a standard protocol as used in our laboratory (24). To remove any residual DNA, the RNA samples were treated with DNase I (Fermentas), and the absence of genomic DNA contamination was confirmed by performing PCR without reverse transcriptase. cDNA synthesis was carried out using 1 µg of total RNA with Qiagen reverse transcriptase, and gene expression was analyzed through PCR amplification using specific primers. Expression of *PfHsp70-1* was assessed using the primers OSB 743 (5′-CAGCTGAAATTGAAACATGTATG-3′) and OSB 220 that amplifies 305 base pair at the 3′ end of the transcript. Similarly, to check the expression of *PfYdj1* and *PfSis1* (PF3D7_0213100), we amplified 350 base pair and 299 base pair of individual transcripts utilizing the primer pairs OSB 742 (5′-ACATGGAGATATTAGAGAAGTTC-3′)-OSB 286 and OSB 748 (5′-ATGATAGATTCCTAAGAGACGC-3′)-OSB 747, respectively. We used OSB94 (5′-CTG TAA CAC ATA ATA GAT CCG AC-3′) and OSB95 (5′-TTA ACC ATC GTT ATC ATC ATT ATT TC-3′) to amplify a 300 bp gene-specific regions of *P. falciparum* aspartate-rich protein (PfARP), as a loading control, that is constitutively expressed in the asexual stage of the parasite (28).

### Western blotting

The protein samples from yeast were prepared using the trichloroacetic acid (TCA) protein extraction method. Parasite proteins were extracted from the ring, trophozoite, and schizont stages by lysing the harvested cells with 0.15% saponin. To prepare RBC protein lysate uninfected human red blood cells (RBCs) were washed three times with PBS and lysed via two rapid freeze–thaw cycles in hypotonic buffer (5  mM Tris-HCl, pH 7.4, containing protease inhibitors), then clarified by centrifugation at 13,000  rpm for 20  min at 4°C. The resulting supernatant containing soluble RBC proteins was collected for SDS–PAGE and immunoblotting. The protein samples were loaded on SDS polyacrylamide gels. A polyvinylidene difluoride (PVDF) membrane was used for transfer, as described previously (26). The primary antibodies anti-GAPDH (Abcam), anti-PfHsp70 (Stress Marq Biosciences), anti-β-Actin (Sigma Aldrich), anti-ScYdj1 (Sigma Aldrich), anti-Gal4BD (Abcam), anti-human R2 (Santa Cruz), anti-PfYdj1 (generated in our lab), and anti-PfSis1 (generated in our lab) were used at 1:5,000 dilutions. Mouse monoclonal anti-DnaK antibody (Abcam, ab69617) was used as the primary antibody at a 1:7,000 dilution. For anti-PfR2 primary antibody, we used 1:500 dilutions. For secondary antibodies, anti-rabbit and anti-mouse antibodies (Thermo Fisher Scientific) were used at 1:10,000 dilutions. The western blots were developed using a chemiluminescent detection system (Bio-Rad).

### Fixed-ratio isobologram method to determine interaction between 116-9e and benzohydroxamate

To investigate the *in vitro* interaction between benzo hydroxamate (Sigma) and 116-9e (Sigma), a fixed-ratio isobologram method was used. The IC_50_ of benzo hydroxamate was determined by treating synchronized ring-stage *P. falciparum* 3D7 parasites with varying concentrations of benzo hydroxamate (1 nM, 10 nM, 100 nM, 1,000 nM, 2 µM, 5 µM, 10 µM, 15 µM, 25 µM, 50 µM, 100 µM, and 200 µM) for 48 h at 37°C. Similarly, the IC_50_ of 116-9e was obtained by exposing synchronized ring-stage parasites to different concentrations of 116-9e (1 nM, 10 nM, 1,000 nM, 10,000 nM, 20 µM, 40 µM, 60 µM, 80 µM, 100 µM) under the same conditions. Parasite growth inhibition was assessed using both Giemsa staining and a SYBR green-based fluorescence assay, measured with a plate reader (Spectramax, M3, Molecular Devices). IC_50_ values were calculated by plotting the percentage of parasite inhibition against drug concentration on a semilogarithmic graph using GraphPad Prism 8. To examine the interaction between benzo hydroxamate and 116-9e, we followed a previously established protocol (29). The two compounds were tested in four fixed ratios (4:1, 3:2, 2:3, and 1:4), with twofold serial dilutions, and their combined effects on parasite growth were assessed in triplicate. Drug assays were performed in 96-well plates, each well containing 200 µL of total reaction volume, comprising 100 µL of parasite culture (1% parasitemia, 5% hematocrit) and 100 µL of medium with or without the drug combination. After incubation at 37°C for 48 h, parasite viability was measured using the SYBR green I-based fluorescence assay, and IC_50_ values for each combination were determined.

The interaction between benzo hydroxamate and 116-9e was analyzed using the fractional inhibitory concentration (FIC) method, where the FIC of each drug was calculated as the ratio of its IC_50_ in the drug mixture to its IC_50_ when used alone. The sum of the FIC values (∑FIC) was used to classify the drug interaction. A ∑FIC value of less than 1 indicated synergism, values between 1 and less than 2 suggested an additive (no interaction) effect, and values of 2 or higher indicated antagonism. The isobologram was generated using GraphPad Prism 8 (24).

### Co-purification assay

We have used a modified protocol obtained from (30). Briefly, Ni-NTA agarose beads were first equilibrated by washing three times with dialysis buffer (20  mM phosphate buffer pH 7.6, 300  mM NaCl, 5% glycerol, and 1 mM DTT). Purified His-tagged PfR2 (10 µg) was then added to the beads and incubated at 4°C for 1 h with gentle rotation to allow binding. Following incubation, the beads were washed three times with wash buffer (20 mM phosphate buffer pH 7.6, 300 mM NaCl) containing 50 mM imidazole to remove unbound protein. We prepared the *P. falciparum* lysate by harvesting synchronized trophozoite-stage parasites and treated with 0.015% saponin, and lysed in lysis buffer obtained from Pierce Crosslink IP Kit (Thermo Fisher Scientific) supplemented with protease inhibitor, and centrifuged at 16,000 × *g* for 20 min at 4°C. The clarified supernatant, containing parasite proteins, was collected, and the total protein concentration was determined using a Nanodrop spectrophotometer (Thermo Fisher Scientific Nanodrop 2000). From the quantified lysate, 200 µg of total protein was incubated with PfR2-bound Ni-NTA beads in binding buffer (20 mM phosphate buffer, pH 7.5, 300 mM NaCl, 0.5% NP-40) at 4°C overnight with continuous rotation to facilitate interaction. Next morning, the beads were washed five times with binding buffer to remove non-specifically bound proteins. The total Ni-NTA beads were resuspended in Laemmli buffer boiled at 95°C for 20 min. The samples were then subjected to SDS-PAGE and transferred onto a PVDF membrane for Western blot analysis. To detect interactions, the membrane was probed with antibodies against PfHsp70, PfYdj1, PfSis1, and PfR2. As controls, two additional conditions were included: Ni-NTA beads were incubated with trophozoite lysate in the absence of PfR2 to assess non-specific binding and Ni-NTA beads bound to PfR2 without parasite lysate were used to check for background signal from the purified protein. A reverse co-purification experiment was performed using the same approach, where instead of PfR2, His-tagged PfYdj1 or its mutant variant Pfydj1^D57N^ were immobilized on Ni-NTA beads and incubated with *P. falciparum* trophozoite lysate to assess its interaction with PfR2.

### Statistical analysis

Statistical analyses were performed using GraphPad Prism 8. Multiple *t*-tests were conducted to compare treated and control groups. A *P*-value of <0.05 was considered statistically significant. Data are presented as mean ± standard deviation (SD) unless stated otherwise. ATP hydrolysis assay data were plotted, and a linear regression model was fitted to determine the relationship between ATP concentration and hydrolysis rate, and the goodness-of-fit (*R*² value) was assessed.

## SUPPLEMENTAL MATERIAL

Fig. S1 mbio.02129-25-s0001.pdf EMBOSS needle pairwise sequence alignment.

Fig. S2 mbio.02129-25-s0002.pdf Validation of antibody specificity and protein purification.

Fig. S3 mbio.02129-25-s0003.pdf Validation of recombinant protein purity and antibody specificity.

Fig. S4 mbio.02129-25-s0004.pdf EMBOSS needle pairwise sequence alignment.

Table S1 mbio.02129-25-s0005.docx Yeast strains.

ASM does not own the copyrights to Supplemental Material that may be linked to, or accessed through, an article. The authors have granted ASM a non-exclusive, world-wide license to publish the Supplemental Material files. Please contact the corresponding author directly for reuse.
