# Supplementary material for: Inhibition of the assembly of Plasmodium Hsp70-1 and Hsp40 complex blocks DNA replication by destabilizing ribonucleotide reductase subunit-2
Source: mBio. 2025 Sep 12;16(10):e02129-25. doi: 10.1128/mbio.02129-25 (PMC12505967; doi:10.1128/mbio.02129-25)
Supplement: Fig. S1 — EMBOSS needle pairwise sequence alignment. [file mbio.02129-25-s0001.pdf]

S1 A.

```
PfYdj1  148 --------------------------------------CTNCEGHGGPKDAKVDCKQCNGRGTKTYMRYHSSVLHQTEVTCNTCRGKGKIFNEKDKCANC 210
                                                  |..|||.||.|.|...|..|||:|.|...|....::.:.:..|:.|.|.|.|.:.||:|.:|
ScYdj1  104 QRPRGPQRGKDIKHEISASLEELYKGRTAKLALNKQILCKECEGRGGKKGAVKKCTSCNGQGIKFVTRQMGPMIQRFQTECDVCHGTGDIIDPKDRCKSC 204
PfYdj1  211 KGMCVLKTRKIIEVYIPKGAPNKHKIVFNGEADEKPNVITGNLVVILNEKQHPVFRREGIDLFMNYKISLYESLTGFVAEVTHLDERKILVNCTNSGFIR 310
            .|..|...|||:||::..|..:..:|||.||||:.|:||.|::|.|::|:.|..|:|:|.||....:|.|..::.|....:.|:....:.|.......|.
ScYdj1  205 NGKKVENERKILEVHVEPGMKDGQRIVFKGEADQAPDVIPGDVVFIVSERPHKSFKRDGDDLVYEAEIDLLTAIAGGEFALEHVSGDWLKVGIVPGEVIA 304
PfYdj1  311 HGDIREVLD-EGMPTYKDPFKKGNLYITFEVEYPMDLIITNENKEVLKILKKQNE---------VEKKYDLENSELEVVSCSPVDKEYIKVRVTKQQQQQ 400
            .| :|:|:: :|||..|.. ..|||.|.|.:::|.:...:.||      |||..|         :.||..::  |..:....|.  :|.:.|.::....
ScYdj1  305 PG-MRKVIEGKGMPIPKYG-GYGNLIIKFTIKFPENHFTSEEN------LKKLEEILPPRIVPAIPKKATVD--ECVLADFDPA--KYNRTRASRGGAN- 392
PfYdj1  401 QQEAYDDEDHQPEMEGGRVACAQQ
                ||.::.:...||  |.||.|
ScYdj1  393 ----YDSDEEEQGGEG--VQCASQ
```

S1 B.

```
PfYdj1    1 MFFSSGFPFDSMGGQQARRKREVNNNKFYEVLNLKKNCTTDEVKKAYRKLAIIHHPDKGGD-------PEKFKEISRAYEVLSDEEKRKLYDE--   86
                                   ....:|.:|.:.::|||:::||||||||::.||||..|       .||||.|:.||:||:||||||:||.
PfSis1    1 -----------------------MGKDYYSILGVSRDCTTNDLKKAYRKLAMMWHPDKHNDEKSKKEAEEKFKNIAEAYDVLADEEKRKIYDTYG   72
```

FIG. S1. EMBOSS Needle Pairwise Sequence Alignment (PSA). **(A)** Pairwise alignment of the C-terminal domain (CTD) between *Plasmodium falciparum* Ydj1 (*PfYdj1*) and *Saccharomyces cerevisiae* Ydj1 (*ScYdj1*), showing 43.5% sequence similarity. **(B)** Pairwise alignment of the J-domain between *PfYdj1* and *Plasmodium falciparum* Sis1 (*PfSis1*), showing 51.6% sequence similarity.
