# Supplementary material for: Inhibition of the assembly of Plasmodium Hsp70-1 and Hsp40 complex blocks DNA replication by destabilizing ribonucleotide reductase subunit-2
Source: mBio. 2025 Sep 12;16(10):e02129-25. doi: 10.1128/mbio.02129-25 (PMC12505967; doi:10.1128/mbio.02129-25)
Supplement: Fig. S2 — Validation of antibody specificity and protein purification. [file mbio.02129-25-s0002.pdf]

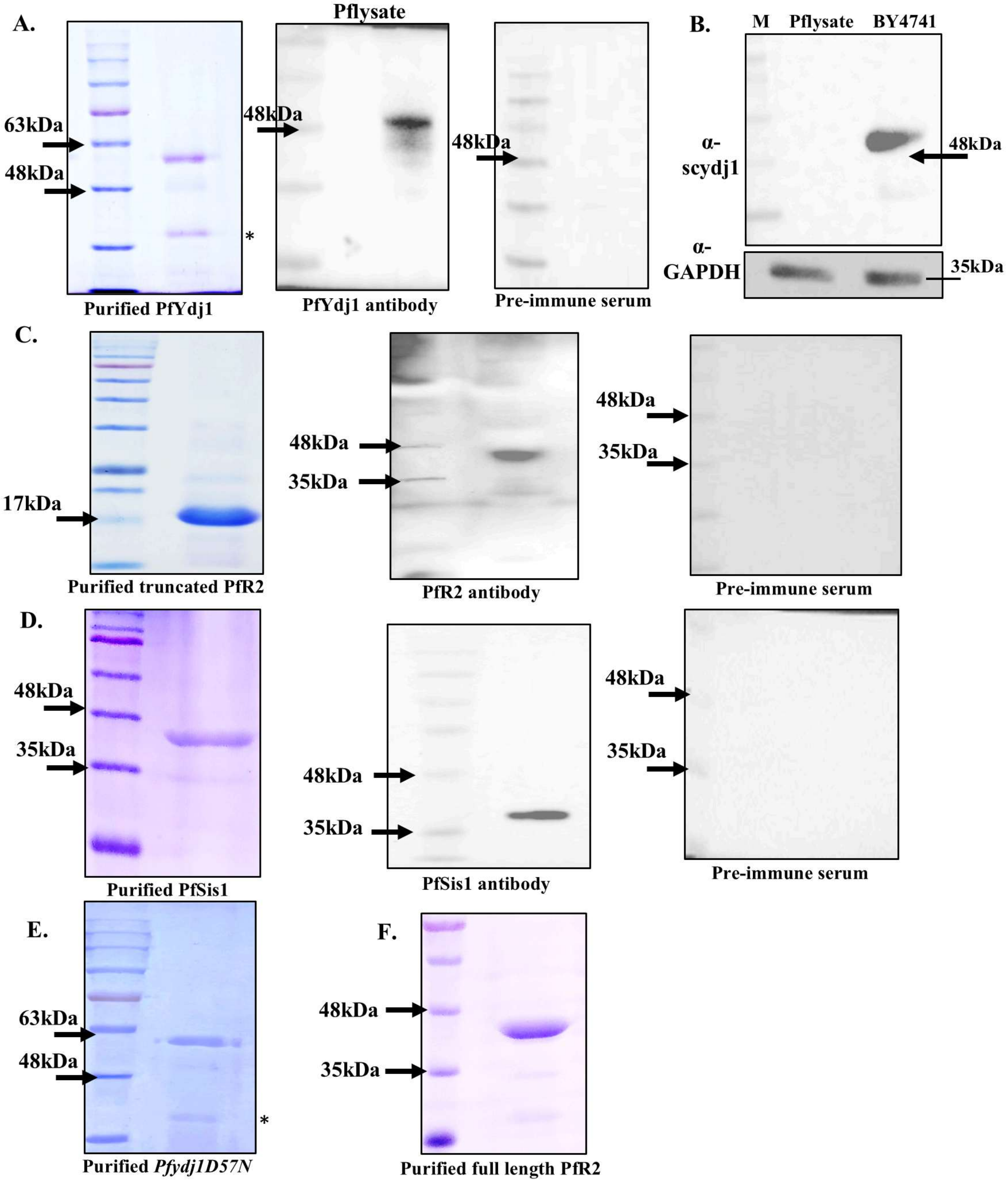

FIG. S2. Validation of antibody specificity and protein purification: (A) SDS-PAGE analysis of purified PfYdj1 (Asterisk denotes a contaminating protein), visualized by Coomassie staining along with immunoblot analysis using anti-PfYdj1 antibodies on Pflysate. Pre-immune serum probing on the same sample is included as a control. Antibodies were generated by gel extraction of the protein to eliminate impurities. **(B)** Western blot showing no cross-reactivity of anti-ScYdj1 antibody with *P. falciparum* lysate. Lysates from *P. falciparum* (trophozoites stage) and *S. cerevisiae* BY4741 were probed with anti-ScYdj1. A ~48 kDa band appeared only in yeast, confirming no cross-reactivity with PfYdj1. GAPDH was used as a loading control. **(C)** Purification of truncated PfR2 and subsequent immunoblot analysis with anti-PfR2 antibodies in early and late trophozoite stage parasite samples, alongside pre-immune serum probing. **(D)** SDS-PAGE analysis of purified PfSis1, followed by immunoblotting with anti-PfSis1 antibodies on Pflysate. Pre-immune serum probing serves as a control. **(E)** SDS-PAGE analysis of purified Pfydj1D57N (Asterisk denotes a contaminating protein). **(F)** SDS-PAGE analysis of purified full-length PfR2.
