# Supplementary material for: Inhibition of the assembly of Plasmodium Hsp70-1 and Hsp40 complex blocks DNA replication by destabilizing ribonucleotide reductase subunit-2
Source: mBio. 2025 Sep 12;16(10):e02129-25. doi: 10.1128/mbio.02129-25 (PMC12505967; doi:10.1128/mbio.02129-25)
Supplement: Fig. S3 — Validation of recombinant protein purity and antibody specificity. [file mbio.02129-25-s0003.pdf]

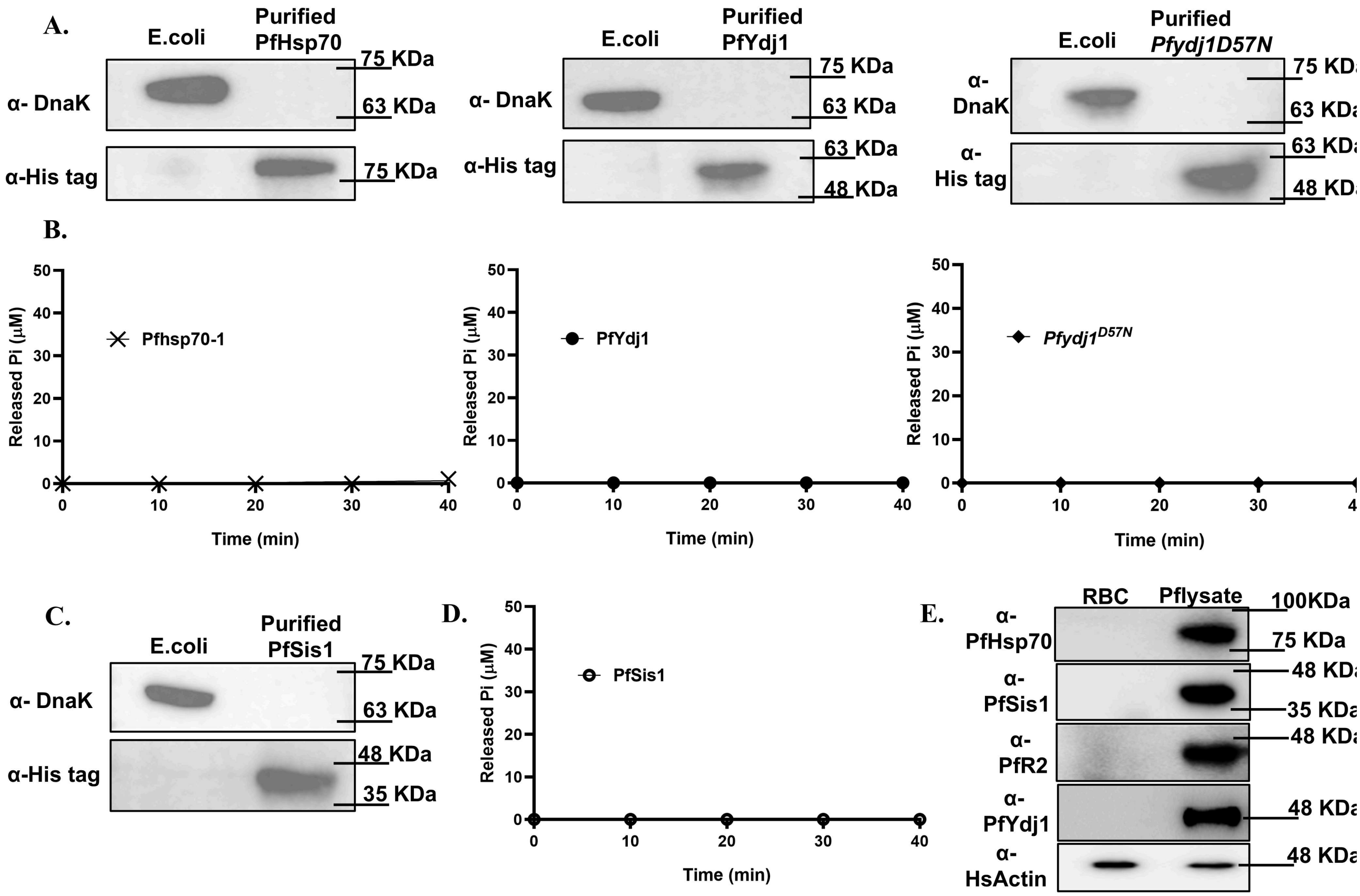

FIG S3. Validation of recombinant protein purity and antibody specificity. **(A)** Western blot analysis using anti-DnaK antibody to assess *E. coli* DnaK contamination in purified PfHsp70, PfYdj1, and *Pfydj1*^{D57N}. *E. coli* lysate was included as a positive control. The same purified protein samples were probed with anti-His tag antibody to confirm the level of His-tagged protein expression, demonstrating that purified protein lacks DnaK contamination **(B)** ATPase activity assays of purified PfHsp70, PfYdj1, and *Pfydj1*^{D57N} were performed in the absence of ATP to confirm no residual ATPase activity or bound ATP in the preparations. **(C, D)** Similar DnaK blot and ATPase assay validation for purified PfSis1. **(E)** Antibody specificity was confirmed by Western blot using lysates from *P. falciparum* (Trophozoites stage) and uninfected red blood cells (RBCs). All antibodies (anti-PfHsp70, anti-PfSis1, anti-PfR2, and anti-PfYdj1) recognized their targets in parasite lysate only, with no cross-reactivity to host proteins. Host Actin served as a loading control.
