# Supplementary material for: Inhibition of the assembly of Plasmodium Hsp70-1 and Hsp40 complex blocks DNA replication by destabilizing ribonucleotide reductase subunit-2
Source: mBio. 2025 Sep 12;16(10):e02129-25. doi: 10.1128/mbio.02129-25 (PMC12505967; doi:10.1128/mbio.02129-25)
Supplement: Fig. S4 — EMBOSS needle pairwise sequence alignment. [file mbio.02129-25-s0004.pdf]

S4 A.

```
PfYdj1 219 -----------------------------------------------------RKIIEVYIPKGAPNKHKIVFNGEADE-KPNVITGNLVVILNEKQHPV 264
                                                                ...:.:.:..|..:..||.|.||.|: .|....|:||..:..|.|..
PfSis1 133 RPSTTTNINTNNYNKPATYEVPLSLSLEELYSGCKKKLKITRKRFMGTKSYEDDNYVTIDVKAGWKDGTKITFYGEGDQLSPMAQPGDLVFKVKTKTHDR 232
PfYdj1 265 FRREGIDLFMNYKISLYESLTGFVAEVTHLDERKILVNCTNSGFIRHGDI------REVLDEGMPTYKDPFKKGNLYITFEVEYPMDLIITNENKEVLKI 358
           |.|:...|.....:.|.::||||...|..||.|.|.|        |..||      :.|..||||:.|.|..||:|.:.|::.:|..|  |:|.|::::.
PfSis1 233 FLRDANHLIYKCPVPLDKALTGFQFIVKSLDNRDINV--------RVDDIVTPKSRKIVAKEGMPSSKYPSMKGDLIVEFDIVFPKSL--TSEKKKIIRE 322
PfYdj1 359 LKKQNEVEKKYDLENSELEVVSCSPVDKEYIKVRVTKQQQQQQQEAYDDEDHQPEMEGGRVACAQQ
           ......
PfSis1 323 TLANTF------------------------------------------------------------
```

FIG. S4. EMBOSS Needle Pairwise Sequence Alignment (PSA). **(A)** Pairwise alignment of the C-terminal domain (CTD) between *Plasmodium falciparum* Ydj1 (*PfYdj1*) and *Plasmodium falciparum* Sis1 (*PfSis1*), showing 25.9% sequence similarity.
