# Supplementary material for: Inhibition of the assembly of Plasmodium Hsp70-1 and Hsp40 complex blocks DNA replication by destabilizing ribonucleotide reductase subunit-2
Source: mBio. 2025 Sep 12;16(10):e02129-25. doi: 10.1128/mbio.02129-25 (PMC12505967; doi:10.1128/mbio.02129-25)
Supplement: Table S1 — Yeast strains. [file mbio.02129-25-s0005.docx]

Supplementary Materials:

**Table S1: Yeast strains used in this study**

| **Strain** | **Genotype** | **Source** |
| --- | --- | --- |
| *BY4741* | *MATa his3Δ1 leu2Δ0 met15Δ0 ura3Δ0* | (26) |
| *Δydj1* | *MATa his3Δ1 leu2Δ0 met15Δ0 ura3Δ0 ydj1::kanMX* | A gift from Prof. K. Mishra, UOH |
| *IAY1* | *MATa his3Δ1 leu2Δ0 met15Δ0 ura3Δ0 ydj1::kanMX [pRS313/Empty]* | This Study |
| *IAY2* | *MATa his3Δ1 leu2Δ0 met15Δ0 ura3Δ0 ydj1::kanMX [pRS313/Scydj1]* | This Study |
| *IAY3* | *MATa his3Δ1 leu2Δ0 met15Δ0 ura3Δ0 ydj1::kanMX [pRS313/PfYdj1]* | This Study |
| *IAY4* | *MATa his3Δ1 leu2Δ0 met15Δ0 ura3Δ0 ydj1::kanMX [pRS313/Pfydj1D57N]* | This Study |
| *IAY5* | *MATa his3Δ1 leu2Δ0 met15Δ0 ura3Δ0 ydj1::kanMX [pRS313/ScSis1]* | This Study |
| *IAY6* | *MATa his3Δ1 leu2Δ0 met15Δ0 ura3Δ0 ydj1::kanMX [pRS313/PfSis1]* | This Study |
| *IAY7* | *MATa his3Δ1 leu2Δ0 met15Δ0 ura3Δ0 ydj1::kanMX [pRS313/ScYdj1][pLA/PfR2]* | This Study |
| *IAY8* | *MATa his3Δ1 leu2Δ0 met15Δ0 ura3Δ0 ydj1::kanMX [pRS313/PfYdj1][pLA/PfR2]* | This Study |
| *IAY9* | *MATa his3Δ1 leu2Δ0 met15Δ0 ura3Δ0 ydj1::kanMX [pRS313/Pfydj1D57N][pLA/PfR2]* | This Study |
| *PJ69-4A* | *MATa trpl-901 leu2-3,112 ura3-52 his3-200 ga14∆ ga180∆ LYS2:: GAL1-HIS3 GAL2-ADE2 met2::GAL7-lacZ* | (23) |
| *IAY10* | *MATa trpl-901 leu2-3,112 ura3-52 his3-200 ga14∆ ga180∆ LYS2:: GAL1-HIS3 GAL2-ADE2 met2::GAL7-lacZ pGBDUC1 pGADC1* | This Study |
| *IAY11* | *MATa trpl-901 leu2-3,112 ura3-52 his3-200 ga14∆ ga180∆ LYS2:: GAL1-HIS3 GAL2-ADE2 met2::GAL7-lacZ pGBDUC1/PfYdj1 pGADC1* | This Study |
| *IAY12* | *MATa trpl-901 leu2-3,112 ura3-52 his3-200 ga14∆ ga180∆ LYS2:: GAL1-HIS3 GAL2-ADE2 met2::GAL7-lacZ pGBDUC1 pGADC1/PfHsp70-1* | This Study |
| *IAY13* | *MATa trpl-901 leu2-3,112 ura3-52 his3-200 ga14∆ ga180∆ LYS2:: GAL1-HIS3 GAL2-ADE2 met2::GAL7-lacZ pGBDUC1/PfYdj1 pGADC1/PfHsp70-1* | This Study |
| *IAY14* | *MATa trpl-901 leu2-3,112 ura3-52 his3-200 ga14∆ ga180∆ LYS2:: GAL1-HIS3 GAL2-ADE2 met2::GAL7-lacZ pGBDUC1/Pfydj1D57N pGADC1* | This Study |
| *IAY15* | *MATa trpl-901 leu2-3,112 ura3-52 his3-200 ga14∆ ga180∆ LYS2:: GAL1-HIS3 GAL2-ADE2 met2::GAL7-lacZ pGBDUC1/Pfydj1D57N pGADC1/PfHsp70-1* | This Study |
| *IAY16* | *MATa trpl-901 leu2-3,112 ura3-52 his3-200 ga14∆ ga180∆ LYS2:: GAL1-HIS3 GAL2-ADE2 met2::GAL7-lacZ pGBDUC1 pGADC1/PfR2* | This Study |
| *IAY17* | *MATa trpl-901 leu2-3,112 ura3-52 his3-200 ga14∆ ga180∆ LYS2:: GAL1-HIS3 GAL2-ADE2 met2::GAL7-lacZ pGBDUC1/PfYdj1 pGADC1/PfR2* | This Study |
| *IAY18* | *MATa trpl-901 leu2-3,112 ura3-52 his3-200 ga14∆ ga180∆ LYS2:: GAL1-HIS3 GAL2-ADE2 met2::GAL7-lacZ pGBDUC1/PfSis1 pGADC1* | This Study |
| *IAY19* | *MATa trpl-901 leu2-3,112 ura3-52 his3-200 ga14∆ ga180∆ LYS2:: GAL1-HIS3 GAL2-ADE2 met2::GAL7-lacZ pGBDUC1/PfSis1 pGADC1/PfR2* | This Study |
